# Iodoquinoline-Biofortified Lettuce as a Safe and Bioavailable Dietary Iodine Source: In Vivo Study in Rats

**DOI:** 10.3390/nu18010036

**Published:** 2025-12-21

**Authors:** Agnieszka Dyląg, Piotr Pawlicki, Anna Gałuszka, Sylwester Smoleń, Aneta Koronowicz

**Affiliations:** 1 Department of Human Nutrition and Dietetics, Faculty of Food Technology, University of Agriculture in Krakow, ul. Balicka 122, 31-149 Krakow, Poland; 2 Department of Basic Sciences, Faculty of Veterinary Medicine, University of Agriculture in Krakow, Redzina 1c, 30-248 Krakow, Poland; piotr.pawlicki@urk.edu.pl (P.P.); anna.galuszka@urk.edu.pl (A.G.); 3 Department of Plant Biology and Biotechnology, Faculty of Biotechnology and Horticulture, University of Agriculture in Krakow, al. Mickiewicza 21, 31-120 Krakow, Poland; sylwester.smolen@urk.edu.pl

**Keywords:** iodine biofortification, *Lactuca sativa* L., iodoquinolines, potassium iodate, thyroid function, nutritional safety, iodine bioavailability, Wistar rats, in vivo study

## Abstract

**Background/Objectives**: Iodine plays a key role in thyroid hormone synthesis and metabolic regulation in vertebrates. This study aimed to evaluate the in vivo bioavailability of iodine and assess selected biochemical parameters and thyroid-related gene expression in male Wistar rats fed lettuce (*Lactuca sativa* L.) biofortified with iodoquinolines (8-hydroxy-7-iodo-5-quinolinesulfonic acid or 5,7-diiodo-8-quinolinol) or potassium iodate. **Methods**: Two iodine intake levels were applied, a nutritionally adequate iodine level and a supranutritional level, to evaluate the nutritional safety of iodine obtained from biofortified vegetables. **Results**: A diet containing lettuce biofortified with iodoquinolines at the adequate level had no significant effect on thyroid hormone concentrations, the expression of *Dio1*, *Dio2*, *Slc5a5*, and *Tpo* genes, or thyroid morphology. While supranutritional iodine intake led to increased levels of T4, fT4, T3, and fT3, all hormone concentrations remained within the physiological range. No elevation in liver enzyme activity (ALT, AST, ALP) was observed, indicating the absence of hepatotoxic effects from high-iodine diets based on biofortified lettuce. Compared to potassium iodate, iodoquinolines demonstrated superior bioavailability, as evidenced by enhanced iodine accumulation in tissues and more efficient thyroid hormone synthesis. **Conclusions**: To the best of our knowledge, this is the first in vivo nutritional study assessing the physiological effects of supranutritional iodine intake from a biofortified plant source. These findings confirm the nutritional safety and efficacy of iodine biofortification using iodoquinolines and highlight the need for further research, including human nutritional clinical trials.

## 1. Introduction

Iodine in various chemical forms is absorbed and utilized by nearly all living organisms, and in chordates it plays the role of an essential micronutrient [1]. In vertebrates, this element is a component of thyroid hormones (3,5,3′,5′-tetraiodo-L-thyronine, T4, and 3,5,3′-triiodo-L-thyronine, T3), and also plays a significant role in energy metabolism, thermoregulation, and physical and mental development [2]. The adverse effects of insufficient iodine intake are well documented in the scientific literature and are most commonly referred to as iodine deficiency disorders (IDDs) [3].

Although iodine deficiency is widely recognized as a public health concern, excessive intake of this micronutrient can also have adverse health effects. The recommended daily iodine intake for healthy adults is approximately 150 µg/day, while the tolerable upper intake level is 1100 µg/day according to WHO and EFSA guidelines [4]. Adults who consume iodine above the tolerable upper intake level may be at increased risk of thyroid dysfunction, including hyperthyroidism, hypothyroidism, and autoimmune thyroiditis [5,6]. Its excessive intake may also contribute to abnormal serum levels of thyroid-stimulating hormone (TSH) and/or thyroid hormones (T3 and T4), raising the risk of thyroid gland disorders. Most studies on the health effects of high iodine intake focus on thyroid function. However, recent research suggests that long-term exposure to high iodine levels may also lead to elevated blood glucose, increased blood pressure, and impact lipid metabolism [7]. Therefore, both insufficient and excessive intakes of this micronutrient should be considered when designing strategies aimed at improving its supply.

Iodized salt, considered a primary tool to combat iodine deficiency, is not widely used in the food industry due to concerns about iodine loss during processing, storage, and transportation [8]. A well-balanced diet can provide adequate iodine for most healthy adults. However, many populations still fail to meet recommended intake levels due to limited use of iodized salt, regional variability in soil iodine content, and specific dietary habits [9]. Therefore, biofortification of vegetables serves as a complementary strategy to traditional dietary recommendations, offering a reliable and highly bioavailable source of iodine, particularly for populations at increased risk of deficiency. This approach does not replace healthy dietary patterns but helps ensure sufficient iodine intake in situations where conventional sources may be inadequate [10].

Iodine intake shows substantial global variability. According to WHO and EFSA surveillance data, median urinary iodine concentrations (UIC) indicate adequate iodine status in many Asian and American populations, whereas mild to moderate deficiency persists across numerous European countries, where UIC values frequently fall below 100 µg/L. The highest prevalence of iodine deficiency is observed in Central and Eastern Europe, South Asia, and Sub-Saharan Africa, reflecting disparities in dietary patterns and inconsistent implementation of salt iodization programs. These geographical differences further underscore the need for alternative dietary strategies, such as plant biofortification, to ensure adequate iodine intake in populations at risk of its deficiency [11].

Nevertheless, for biofortified plants to effectively improve nutritional status, it is essential to provide forms of iodine with high bioavailability. While iodine bioavailability from most food products is generally high (>90%), metabolic processes occurring within the plant can influence the final chemical form of iodine and its efficiency of utilization by the organism [12,13,14]. Considering that the regulated synthesis of thyroid hormones depends on adequate iodine availability, the bioavailability of iodine in biofortified plants must be assessed prior to implementing such interventions. This assessment is a key factor in predicting the effectiveness of biofortification programs, as it allows determination of the minimum iodine content in the plant required to effectively improve the nutritional status of a given population [15].

In vivo studies, both in animals and humans, provide a more comprehensive understanding of how biofortified plants influence mineral absorption and overall nutritional status. Animal models enable analysis of micronutrient absorption throughout the organism, as well as assessment of potential changes in gene expression and the functioning of organs and tissues resulting from consumption of fortified plants, which is crucial for evaluating the health safety of their potential consumers [14,16].

The aim of the present study is to evaluate the bioavailability of iodine and to analyze selected biochemical parameters and gene expression related to thyroid hormone synthesis and metabolism in Wistar rats fed a diet supplemented with lettuce biofortified with iodoquinolines (8-hydroxy-7-iodo-5-quinolinesulfonic acid or 5,7-diiodo-8-quinolinol) or potassium iodate. In each analysis, the effects of two levels of iodine intake delivered with lettuce were compared—the recommended dose (in accordance with the control AIN-93G diet) and a dose twice as high—in order to assess whether excessive iodine intake affects the analyzed parameters.

8-hydroxy-7-iodo-5-quinolinesulfonic acid and 5,7-diiodo-8-quinolinol were selected as iodine donors due to their good water solubility, high chemical stability, and efficient iodine accumulation in hydroponically grown lettuce. In the plant, most of the iodoquinoline is converted into iodide (I^−^), which represents an easily available form of iodine for the consumer, whereas the metabolic fate of the small fraction of untransformed organoiodine residues has not yet been fully elucidated. Compared with potassium iodate, the use of these donors also promotes higher accumulation of bioactive compounds, such as polyphenols, vitamin C, and B vitamins. These components may further support iodine utilization and enhance the nutritional and health-promoting value of the plant, while maintaining a favorable safety profile for the potential consumer [17].

## 2. Materials and Methods

### 2.1. Plant Material and Growing Conditions

Lettuce (*Lactuca sativa* L. var. *capitata*) cv. ‘Melodion’ was grown hydroponically using the Nutrient Film Technique (NFT). The experiment was conducted in spring 2021 in a greenhouse at the Faculty of Biotechnology and Horticulture, University of Agriculture in Kraków, Poland (50°05′04.1″ N 19°57′02.1″ E). The seeds were sown in mid-February. Seedlings with 4–5 true leaves (mid-March) were transplanted into the NFT system and placed in styrofoam slabs filling NFT beds (holes spaced 25 cm apart)—a “dry hydroponic” method of cultivation without substrate.

Organic iodine compounds (iodoquinolines) and an inorganic form of this element (potassium iodate) were introduced into the nutrient solution. The following treatments were analyzed: (a) control (no iodine application), (b) KIO_3_, (c) 8-hydroxy-7-iodo-5-quinolinesulfonic acid, and (d) 5,7-diiodo-8-quinolinol. All substances were applied at a concentration of 5 µM as molar mass equivalents of each compound. The nutrient solution was applied continuously throughout the entire cultivation period until harvest.

Lettuce plants were harvested at the beginning of May, when they reached the rosette stage with 5–6 fully expanded true leaves. Additional information regarding the plant material and growing conditions is provided in our earlier publication [17].

#### Chemical Composition Analysis of Plant Material

The basic chemical composition of the plant material included in the animals’ diet was determined, i.e., protein, crude fat, digestible carbohydrates, dietary fiber, and ash. In addition, dry matter content was measured to standardize the plant material used in the animals’ diet. Total iodine concentration, iodide content (I^−^), quinoline, individual iodoquinolines, selected macro- and microelements (P, S, Na, K, Mg, Ca, Fe, Cu, Zn, Mn, Mo, B), B vitamins (B1, B2, B3, PP, B5, B6, B9), vitamin C (L-ascorbic acid, dehydroascorbic acid), chlorides, nitrogen compounds (NO_3_^−^, NO_2_^−^, NH_4_^+^), total polyphenols, and antioxidant activity were also assessed. The methodology and details concerning the chemical composition analysis of iodine-biofortified lettuce in the form of iodoquinolines were described in a separate publication [17].

### 2.2. Animal Study

The in vivo experiment was conducted on male Wistar rats and lasted 7 weeks. All procedures were approved by the 2nd Local Ethics Committee for Animal Experiments at the Institute of Pharmacology of the Polish Academy of Sciences in Kraków (resolution no. 212/2023, dated 9 November 2023).

#### 2.2.1. Study Design

The study was performed at the Center for Experimental and Innovative Medicine of the University of Agriculture in Kraków. Male Wistar rats, a strain widely recognized as one of the most versatile and effective models in nutritional studies, were used [18]. Four-week-old rats (n = 56), with an average body weight of 125 ± 10 g, were obtained from the internal breeding facility of the Animal House at the Faculty of Pharmacy, Jagiellonian University in Kraków. Before the main part of the experiment, the animals underwent a 7-day acclimatization period to minimize stress associated with the presence of personnel, during which they were fed a standard laboratory diet.

#### 2.2.2. Experimental Protocol

After acclimatization, the rats were allocated to eight experimental groups (n = 7 per group) using simple randomization. Animals were marked at the time of allocation to the respective groups, the assignment was coded, and cages were labeled with the group number. The group size was determined based on standard practices in nutritional studies with Wistar rats, allowing for reliable statistical analysis while minimizing the number of animals used.

During the acclimatization period and the experiment, the rats were kept in conventional cages, with each group housed in four cages (in the first, second, and third—two individuals, and in the fourth—one). The only exceptions were the second and seventh weeks of the experiment, when the animals were housed individually in stainless steel metabolic cages. The animals were kept at a temperature of 22 °C (±2 °C), relative humidity of 55% (±10%), and a 12/12 h light/dark cycle. Environmental enrichment was provided in the cages, including wooden blocks (chew toys), tunnels, wooden houses, and bedding/nesting material to provide shelter and reduce stress and aggressive behaviors.

Throughout the experiment, the rats had ad libitum access to the tested diet and distilled water (tap water could serve as an additional source of iodine). They were also under continuous observation to assess their behavior and general condition. Diet intake was monitored daily, while body weight gain was recorded once a week.

#### 2.2.3. Diets and Supplementations

During the experiment, the animals were fed diets based on the standard AIN-93G formulation [19], modified by the addition of freeze-dried (lyophilized) lettuce. The diets were prepared by ZooLab (Sędziszów, Poland).

The types of diets used in the individual groups were as follows: **Group 1**—AIN-93G diet (control), the mineral mix contained iodine (in the form of KI) in the amount recommended by Reeves [19]; **Group 2**—AIN-93G diet with the addition of lyophilized lettuce not subjected to the iodine biofortification process, the iodine content (as KI) in the mineral mix was the same as in the control diet; **Groups 3**, **4**, **5**—AIN-93G diet (no KI in the mineral mix) with the addition of lyophilized lettuce biofortified with iodine in the form of potassium iodate (Group 3), 8-hydroxy-7-iodo-5-quinolinesulfonic acid (Group 4), and 5,7-diiodo-8-quinolinol (Group 5), providing iodine in the amount recommended for this diet; **Groups 6**, **7**, **8**—AIN-93G diet (no KI in the mineral mix) with the addition of lyophilized lettuce biofortified with iodine in the form of potassium iodate (Group 6), 8-hydroxy-7-iodo-5-quinolinesulfonic acid (Group 7), and 5,7-diiodo-8-quinolinol (Group 8), supplying iodine in an amount twice the recommended level for this diet.

The detailed composition of the experimental diets is presented in Table 1, where the diet number corresponds to the number of the experimental group. In addition, the iodine content in the feeds was determined to further verify the level of this element after diet preparation (Table S1). Iodine levels were analyzed using the ICP-MS/MS (Inductively Coupled Plasma Tandem Mass Spectrometry) technique, according to the procedure described by Smoleń et al. [20].

#### 2.2.4. Sample Collection

Urine and feces were collected over 5 days during the second and seventh weeks of the experiment (days 8–12 and 44–48, respectively) to evaluate iodine excretion (iodine content determination). Moreover, urine samples were standardized in volume before analysis.

At the end of the experiment, the animals were fasted and then anesthetized using sodium pentobarbital (Morbital; Biowet Puławy, Puławy, Poland) at a dose of 1–2 mL kg^−1^ body weight, administered via intraperitoneal injection. Blood was collected by cardiac puncture into tubes without anticoagulant and centrifuged (1500× *g*, 15 min, 4 °C) to obtain serum. Feces were collected directly from the colon during the necropsy. The thyroid, liver, heart, kidneys, and testes were isolated and immediately frozen in dry ice. Part of the thyroid glands was fixed in 10% buffered formalin (Chempur, Piekary Śląskie, Poland) and prepared for histological evaluation. The remaining samples were stored at −80 °C until further analyses. Selected indicators reflecting the functional state of the thyroid gland were determined in blood serum and the gland itself. These indicators included biochemical parameter analysis and gene expression. Furthermore, iodine levels were measured in all dissected organs.

### 2.3. Laboratory Analyses

#### 2.3.1. Assessment of Iodine Content in Urine, Feces, and Selected Organs

Urine samples were thawed and mixed, and 4.8 mL of each sample was transferred to a polypropylene tube. Subsequently, 0.2 mL of tetramethylammonium hydroxide (TMAH) was added. The mixture was incubated, mixed, and diluted 200-fold with triple-distilled water. Iodine content was determined using the previously mentioned ICP-MS/MS method with a triple quadrupole spectrometer (iCAP TQ ICP-MS; Thermo Fisher Scientific, Waltham, MA, USA), in accordance with the PN-EN 15111:2008 standard [21] and the procedure described by Smoleń et al. [20].

Feces and organs were lyophilized (Christ Alpha 1-4 LSC plus, Martin Christ Gefriertrocknungsanlagen GmbH, Osterode am Harz, Germany), then weighed and ground using a laboratory mill (type WŻ-1; ZBPP, Sadkiewicz Instruments, Bydgoszcz, Poland). Samples (with particle sizes of approximately 1 mm) prepared in this manner were analyzed for iodine concentration using the same ICP-MS/MS technique as applied for urine analysis.

#### 2.3.2. Analysis of Biochemical Parameters

The study material consisted of blood serum, except for elastase, which was measured in stool samples. Thyroid hormones analysis, including thyroxine (T4), free thyroxine (fT4), triiodothyronine (T3), and free triiodothyronine (fT3), was performed using the chemiluminescent immunoassay method (CLIA) on an Access 2 analyzer (Beckman Coulter Diagnostics, Brea, CA, USA). Biochemical parameters such as high-density lipoproteins (HDL cholesterol), total cholesterol (TC), triglycerides (TG), alanine aminotransferase (ALT), aspartate aminotransferase (AST), alkaline phosphatase (ALP), urea, amylase, and lipase were measured spectrophotometrically using a semi-automatic analyzer BA-400 (BioSystems Diagnostics Pvt. Ltd., Sriperumbudur, Tamil Nadu, India). In both cases, dedicated reagent kits were used, according to manufacturers’ protocols. Additionally, low-density lipoproteins and very-low-density lipoproteins (LDL + VLDL cholesterol) levels were estimated by calculating the difference between total cholesterol and HDL cholesterol concentrations [22,23]. Thyroid-stimulating hormone (TSH), malondialdehyde (MDA), and elastase concentrations were measured spectrophotometrically (Infinite 200 Pro microplate reader, Tecan Group Ltd., Männedorf, Switzerland) by enzyme-linked immunosorbent assay (ELISA), using the following kits, respectively: Rat TSH ELISA Kit (Cat. No. 812-NSL1179r, Sunlong Biotech Co., Ltd., Hangzhou, Zhejiang, China), Rat MDA ELISA Kit (Cat. No. 812-NSL1352r, Sunlong Biotech Co., Ltd.), and Rat Elastase 3B ELISA Kit (Cat. No. ELK6605, ELK Biotechnology Co., Ltd., Denver, CO, USA).

#### 2.3.3. Gene Expression Analysis

##### RNA Isolation and cDNA Synthesis

Total RNA was isolated from 15 mg of frozen rat thyroid tissue using the AllPrep DNA/RNA Mini Kit (Qiagen, Hilden, Germany; Cat. No. 80204) in accordance with the manufacturer’s protocol. RNA yield and purity were evaluated with a NanoDrop™ 2000/2000c spectrophotometer (Thermo Fisher Scientific, Cat. No. ND-2000). All samples demonstrated A260/A280 ratios ≥ 1.9 and A260/A230 ratios ≥ 2.0, consistent with high-quality RNA. For reverse transcription, 200 ng of total RNA per sample was converted into complementary DNA (cDNA) in a 20 µL reaction using the High-Capacity cDNA Reverse Transcription Kit (Thermo Fisher Scientific, Cat. No. 4368814). Thermal cycling conditions followed the manufacturer’s recommendations: 10 min at 25 °C, 120 min at 37 °C, and 5 min at 85 °C. Following synthesis, cDNA concentration was re-measured using the NanoDrop™ 2000/2000c.

##### Quantitative Real-Time PCR Analysis (qPCR)

Quantitative real-time PCR (qPCR) was performed to assess gene expression levels using TaqMan^®^ Gene Expression Assays (Thermo Fisher Scientific) specific for the following rat genes: *Dio1* (Rn00572183_m1), *Dio2* (Rn00581867_m1), *Slc5a5* (Rn00583900_m1), and *Tpo* (Rn00571159_m1). The expression of 18S rRNA (Rn01531966_m1) was used as an endogenous control for normalization. Each reaction was carried out in a final volume of 10 µL, comprising 1× TaqMan^®^ Gene Expression Master Mix (Thermo Fisher Scientific), 200 nM of the corresponding probe, and 2 µL of cDNA (approximately 100 ng). The concentration and purity of cDNA were verified using a NanoDrop™ 2000/2000c spectrophotometer (Thermo Fisher Scientific). Amplifications were run on a Roche LightCycler^®^ 96 System using the following thermal profile: initial denaturation at 95 °C for 2 min, followed by 40 cycles of denaturation at 95 °C for 15 s and annealing/extension at 60 °C for 15 s. Each sample was analyzed in technical triplicates. No-template controls (NTCs) were included in each run to monitor for contamination. Each experimental group consisted of four biological replicates, with each replicate comprising pooled material from two individuals. Gene expression levels were normalized to 18S rRNA and calculated using the 2^−∆∆Ct^ method. Data were analyzed with LightCycler^®^ 96 Software (version 1.1, Roche Diagnostics, Mannheim, Germany).

#### 2.3.4. Histological Analysis 

Formalin-fixed, paraffin-embedded tissue specimens were sectioned at a thickness of 5 µm and processed for histological evaluation. Standard hematoxylin and eosin (HE) staining was employed to assess overall tissue architecture and cytomorphological features with high resolution. In addition, Masson’s trichrome staining was conducted using a commercially available kit (Abcam, Cambridge, UK) to enable the specific identification and differentiation of collagenous connective tissue components within the extracellular matrix. Histological analyses were performed using a Leica DMR brightfield microscope (Leica Microsystems, Wetzlar, Germany).

### 2.4. Statistical Analysis

Collected data are presented as mean ± standard deviation (SD). Data were tested for normality using the Shapiro–Wilk test, and observations were considered independent, with each animal representing a single experimental unit. Statistical analysis was performed using Statistica software version 13.1 (StatSoft Inc., Tulsa, OK, USA). Differences between groups were assessed using one-way analysis of variance (ANOVA) followed by Tukey’s post hoc test. To compare iodine concentrations in urine and feces between the second and seventh weeks of the experiment within each group, the paired Student’s *t*-test was used. In both tests, results were considered statistically significant at *p* ≤ 0.05.

## 3. Results

### 3.1. Iodine Content

#### 3.1.1. Iodine Content in Urine and Feces

Table 2 presents the iodine content in the urine and feces of rats fed diets supplemented with the tested lettuce.

In the second and seventh weeks of the experiment, an average decrease of 22.08% and 32.71%, respectively, was observed in the iodine content of urine from rats receiving diets containing biofortified lettuce that supplied iodine amounts comparable to the control diet (Groups 3–5). The lowest iodine concentration in urine was observed in rats fed diets supplemented with lettuce enriched with 5,7-diiodo-8-quinolinol (Group 5) and 8-hydroxy-7-iodo-5-quinolinesulfonic acid (Group 4) during the second and seventh weeks of the experiment, respectively. The decrease in iodine levels amounted to 29.20% and 38.16%, respectively, compared to the iodine content in urine of animals on the control diet.

In both experimental weeks, the urine of rats fed diets containing biofortified lettuce providing iodine at twice the recommended dose of the AIN-93G diet showed an average increase in iodine concentration of 15.98% (second week) and 10.31% (seventh week). The highest iodine content in urine during both weeks was recorded in rats from Group 7, which consumed diets supplemented with lettuce enriched with 8-hydroxy-7-iodo-5-quinolinesulfonic acid. This content was higher by 27.27% (second week) and 20.59% (seventh week) compared to animals in Group 1 (control).

The use of the tested diets also contributed to an increase in iodine levels in the feces of rats in both the second and seventh weeks of the experiment (compared to the control diet). This increase averaged 21.88% and 22.62% for groups receiving iodine at the recommended dose in the AIN-93G diet (Groups 3–5) and 46.08% and 63.92% for groups receiving iodine at twice the recommended dose (Groups 6–8).

Additionally, between the second and seventh weeks of the experiment, a significant decrease in urinary iodine levels was observed in rats receiving, through the diet, both the recommended (Groups 3 and 4) and the double (Groups 6–8) iodine amounts. The decrease was 7.28% and 30.10% for the recommended amount, and 5.47%, 5.07%, and 3.53% for the double amount, respectively. In contrast, fecal iodine levels increased in rats receiving the double iodine amount (Groups 6–8) by 9.52%, 16.69%, and 10.06%.

#### 3.1.2. Iodine Content in Selected Organs

The inclusion of biofortified lettuce in the diets led to a number of statistically significant changes in iodine content across selected rat organs (Table 3).

Feeding animals with diets containing lettuce biofortified with iodoquinoline, providing iodine in an amount consistent with AIN-93G dietary recommendations, resulted in an average increase in iodine concentration of 34.23% in the liver, 45.89% in the heart, and 18.89% in the testes. In the thyroid and kidneys of rats whose diets included lettuce enriched with 8-hydroxy-7-iodo-5-quinolinesulfonic acid (Group 4), a 7.26% increase and a 20.13% decrease in iodine content were observed, respectively. In contrast, the iodine levels in these organs of rats receiving lettuce fortified with 5,7-diiodo-8-quinolinol (Group 5) did not differ from those in the control group. Meanwhile, rats consuming lettuce enriched with potassium iodate (inorganic iodine; Group 3) exhibited decreased iodine content in the thyroid, liver, kidneys, and testes by an average of 10.71%, 25.46%, 31.91%, and 37.14%, respectively. No statistically significant changes were found in heart iodine content in this group.

The use of a diet containing lettuce biofortified with iodoquinoline, providing twice the iodine level of the control diet, led to an increase in iodine levels across all examined organs, amounting to an average of 14.70% in the thyroid, 59.91% in the liver, 12.11% in the kidneys, 73.53% in the heart, and 41.21% in the testes. Furthermore, in animals fed lettuce enriched with potassium iodate (Group 6), heart iodine levels increased by 24.01%, while thyroid, liver, and kidney iodine levels showed no statistically significant difference compared to the control group. On the other hand, iodine content in the testes decreased by 31.83%.

Iodine levels in the urine, feces (at both time points), and all tested organs in the group of rats fed non-biofortified lettuce (Group 2; iodine supplied via mineral mix) did not differ significantly from those in the control group (Table 2 and Table 3).

### 3.2. Biochemical Parameters

#### 3.2.1. Hormones Regulating Thyroid Function

The addition of lettuce biofortified with iodine compounds providing the element at levels recommended for the AIN-93G diet differently affected the levels of thyroid hormones in the serum of rats (Table 4). The inclusion of lettuce enriched with potassium iodate in their diet (Group 3) led to decreases in T4, fT4, T3, and fT3 concentrations by 16.14%, 32.52%, 10.77%, and 11.02%, respectively, compared to values obtained in the control group. In contrast, feeding rats a diet containing lettuce biofortified with iodoquinoline (Groups 4 and 5) did not result in statistically significant changes in thyroid hormone levels. The exception was the T4 hormone, which showed an average increase of 10.37%.

The introduction of lettuce enriched with various iodine forms at concentrations twice those recommended for the AIN-93G diet caused a significant increase in all studied thyroid hormones in serum by an average of 14.79% (T4), 35.85% (fT4), 77.95% (T3), and 32.68% (fT3). Only in the serum of rats receiving lettuce fortified with mineral iodine (Group 6) did the thyroxine content not differ significantly from that in Group 1. The highest levels of fT4, T3, and fT3 hormones were observed in the serum of animals fed with lettuce enriched with 5,7-diiodo-8-quinolinol. These levels were higher than those in the control group by 60.59%, 118.46%, and 52.30%, respectively. The highest thyroxine concentration was found in the serum of rats whose diet included lettuce biofortified with both 5,7-diiodo-8-quinolinol and 8-hydroxy-7-iodo-5-quinolinesulfonic acid.

The serum TSH concentration in all experimental groups remained statistically unchanged compared to the control group.

#### 3.2.2. Lipid Profile

Feeding rats with diets containing the tested lettuce had a noticeable impact on their lipid profile (Table 5). In the blood serum of animals from all experimental groups (except for Group 2), a decrease in HDL cholesterol and total cholesterol levels was observed, averaging 22.59% and 20.86% (Groups 3–5), and 31.64% and 23.68% (Groups 6–8), respectively. The inclusion of lettuce fortified with potassium iodate and 5,7-diiodo-8-quinolinol in the diet, providing iodine in the same amount as in the control diet (Groups 3 and 5), resulted in a reduction of LDL + VLDL cholesterol in their serum by 14.58% and 27.99%, respectively. On the other hand, in the serum of animals fed with lettuce enriched with potassium iodate containing iodine at a concentration twice as high as in the AIN-93G diet (Group 6), a 20.02% decrease in these cholesterol fractions was noted. However, the addition of lettuce biofortified with 8-hydroxy-7-iodo-5-quinolinesulfonic acid, providing this element in both the recommended and double amounts (Groups 4 and 7), did not lead to statistically significant changes in LDL + VLDL cholesterol levels in serum. No statistically significant changes were also observed in the group receiving a double dose of iodine with lettuce enriched with 5,7-diiodo-8-quinolinol (Group 8). The triglyceride concentration in the serum of rats from almost all experimental groups did not differ significantly from the control group. Only in the serum of animals consuming lettuce biofortified with 5,7-diiodo-8-quinolinol (Group 5) was a 27.12% decrease in triglyceride levels observed, consistent with the trends seen in the other analyzed lipid profile parameters.

#### 3.2.3. Other Biochemical Parameters

The inclusion of the tested lettuce in the diets resulted in a decrease in amylase and urea concentrations in the blood serum of rats from all experimental groups by an average of 18.70% and 5.42% (Group 2), 23.97% and 22.01% (Groups 3–5), and 12.34% (Groups 6–8) and 14.15% (Groups 6 and 8), respectively (Table 6). On the other hand, in the serum of animals from Group 7, receiving feed supplemented with lettuce fortified with 8-hydroxy-7-iodo-5-quinolinesulfonic acid (iodine at twice the dose recommended in the AIN-93G diet), urea levels did not differ significantly compared to the control group. MDA concentration in this group was also not significantly altered. Furthermore, in the serum of rodents from Groups 3–5, whose diet included lettuce supplying iodine at levels equivalent to the control diet, ALT and ALP levels decreased on average by 16.02% and 18.24%, respectively (Table 6). In rats fed diets containing lettuce enriched with 5,7-diiodo-8-quinolinol (Group 5), ALP levels showed no significant change. Similarly, AST, lipase, and MDA serum concentrations in animals from Groups 3–5 were not significantly altered (Table 6).

Conversely, the use of lettuce biofortified with iodine at twice the recommended AIN-93G dose led to an increase in enzyme concentrations such as ALP and lipase by an average of 28.83% and 25.46%, respectively. Only in the serum of rats consuming feed supplemented with lettuce fortified with 5,7-diiodo-8-quinolinol (Group 8) were ALP levels statistically unchanged compared to the control group. AST levels did not differ significantly in Group 7 or in the previously mentioned group, while Group 6 showed an 11.59% increase. Moreover, Groups 6 and 8 exhibited decreases in MDA levels of 13.75% and 13.48%, respectively. The inclusion of lettuce supplying iodine at twice the control group’s concentration differently affected serum ALT levels depending on the iodine form used for biofortification (Table 6). The addition of lettuce enriched with potassium iodate caused a 23.76% increase in ALT, while lettuce biofortified with 5,7-diiodo-8-quinolinol led to a 13.44% decrease. Lettuce enriched with 8-hydroxy-7-iodo-5-quinolinesulfonic acid had no significant effect on ALT levels.

In the serum of animals fed with non-biofortified lettuce (Group 2), the concentrations of all analyzed biochemical parameters (except for amylase and urea) remained unchanged (Table 4, Table 5 and Table 6).

### 3.3. Relative Expression of Selected Genes Involved in Thyroid Hormone Metabolism

**Figure 1:**
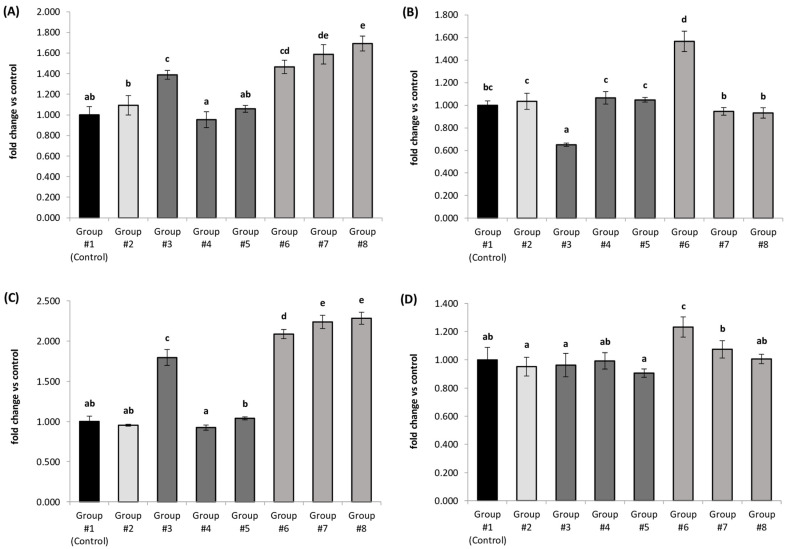
Relative expression of (**A**) *Dio1*, (**B**) *Dio2*, (**C**) *Slc5a5* (NIS), and (**D**) *Tpo* genes in the thyroid gland of experimental rats. Results are presented as mean ± standard deviation (n = 7). Bars marked with different letters differ significantly at *p* ≤ 0.05. Description of experimental groups: **Group #1**—rats fed the AIN-93G (control) diet; **Group #2**—rats fed the AIN-93G diet with the addition of lyophilized non-biofortified lettuce, with KI from the mineral mixture providing iodine in the amount recommended for this diet; **Group #3**, **#4**, **#5**—rats fed the AIN-93G diet with the addition of lyophilized iodine-biofortified lettuce in the form of potassium iodate (Group #3), 8-hydroxy-7-iodo-5-quinolinesulfonic acid (Group #4), and 5,7-diiodo-8-quinolinol (Group #5), providing iodine in the amount recommended for this diet; **Group #6**, **#7**, **#8**—rats fed the AIN-93G diet with the addition of lyophilized iodine-biofortified lettuce in the form of potassium iodate (Group #6), 8-hydroxy-7-iodo-5-quinolinesulfonic acid (Group #7), and 5,7-diiodo-8-quinolinol (Group #8), providing iodine in an amount twice that recommended for this diet.

In the thyroid glands of rats fed a diet containing lettuce biofortified with iodoquinoline (Groups 4 and 5), providing iodine at levels equivalent to the AIN-93G diet, no significant differences were observed in the mRNA levels of all analyzed genes: *Dio1*, *Dio2*, *Slc5a5* (NIS), and *Tpo*. Conversely, rats receiving a diet with lettuce enriched with potassium iodate (Group 3) were observed to have an up-regulation of *Dio1* and *Slc5a5* mRNA by 39% and 80%, respectively, and a down-regulation of *Dio2* mRNA by 35%. The expression of *Tpo* showed no statistically significant difference compared to the control group (Figure 1).

A twofold higher iodine intake from fortified lettuce triggered several significant alterations in gene expression related to thyroid hormone metabolism and synthesis. Animals consuming lettuce biofortified with organic iodine forms (Groups 7 and 8) were found to have up-regulation of *Dio1* (by an average of 64%) and *Slc5a5* (by an average of 126%) transcripts. The mRNA levels of *Dio2* and *Tpo* in these groups remained statistically unchanged compared to controls. In the thyroids of rats fed lettuce enriched with inorganic iodine (Group 6), increased transcriptional activity was noted not only for *Dio1* and *Slc5a5* but also for *Dio2* and *Tpo*. Specifically, mRNA expression of *Dio1*, *Dio2*, *Slc5a5*, and *Tpo* increased by 47%, 57%, 109%, and 23%, respectively, compared to the control group (Figure 1).

Additionally, in animals receiving a diet containing non-biofortified lettuce, where iodine was supplied via a mineral mix (Group 2), the gene expression profile was similar to that of the control group (Figure 1).

### 3.4. Histological Evaluation

In the histological assessment of the thyroid glands of rats from all groups, a variation in the size and distribution of follicles was observed, along with the presence of colloid that was either homogeneous or occasionally foamy. Focal detachment of thyrocytes from the follicular lumen was sporadically noted, while the structure of collagen fibers remained preserved. Only in the group of rats receiving lettuce biofortified with potassium iodate, providing twice the recommended amount of iodine, were mild morphological changes observed. These changes included a predominance of large and medium-sized follicles in the central region, disruption of the typical zonal arrangement, and slight, localized fibrosis.

#### 3.4.1. Hematoxylin and Eosin Staining

**Figure 2:**
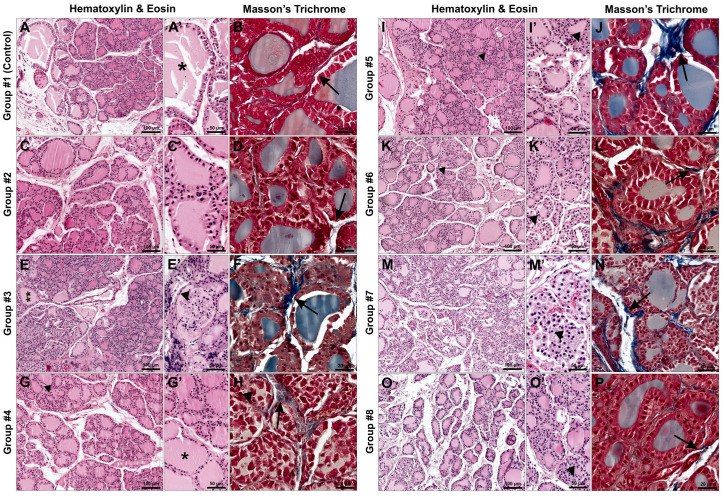
Histological images of rat thyroid stained with hematoxylin and eosin (H&E) and Masson’s trichrome. Scale bars: H&E—100 µm and 50 µm; Masson’s trichrome—20 µm. Description of subfigures: (**A**) H&E, 100 µm, (**A′**) H&E, 50 µm, (**B**) Masson’s trichrome—Group #1; (**C**) H&E, 100 µm, (**C′**) H&E, 50 µm, (**D**) Masson’s trichrome—Group #2; (**E**) H&E, 100 µm, (**E′**) H&E, 50 µm, (**F**) Masson’s trichrome—Group #3; (**G**) H&E, 100 µm, (**G′**) H&E, 50 µm, (**H**) Masson’s trichrome—Group #4; (**I**) H&E, 100 µm, (**I′**) H&E, 50 µm, (**J**) Masson’s trichrome—Group #5; (**K**) H&E, 100 µm, (**K′**) H&E, 50 µm, (**L**) Masson’s trichrome—Group #6; (**M**) H&E, 100 µm, (**M′**) H&E, 50 µm, (**N**) Masson’s trichrome—Group #7; (**O**) H&E, 100 µm, (**O′**) H&E, 50 µm, (**P**) Masson’s trichrome—Group #8. Description of experimental groups: **Group #1**—rats fed the AIN-93G (control) diet; **Group #2**—rats fed the AIN-93G diet with the addition of lyophilized non-biofortified lettuce, with KI from the mineral mixture providing iodine in the amount recommended for this diet; **Group #3**, **#4**, **#5**—rats fed the AIN-93G diet with the addition of lyophilized iodine-biofortified lettuce in the form of potassium iodate (Group #3), 8-hydroxy-7-iodo-5-quinolinesulfonic acid (Group #4), and 5,7-diiodo-8-quinolinol (Group #5), providing iodine in the amount recommended for this diet; **Group #6**, **#7**, **#8**—rats fed the AIN-93G diet with the addition of lyophilized iodine-biofortified lettuce in the form of potassium iodate (Group #6), 8-hydroxy-7-iodo-5-quinolinesulfonic acid (Group #7), and 5,7-diiodo-8-quinolinol (Group #8), providing iodine in an amount twice that recommended for this diet. Legend: ⁎—foamy collagen structure; →—single collagen fiber; ▼—detachment of thyrocytes into the lumen; ⁑—single large follicles in the central zone.

The thyroids of rats from the control group and the group receiving a diet containing non-biofortified lettuce (with iodine provided by a mineral mix) exhibited preserved architecture, with a clear division of follicles into peripheral and central zones—larger follicles were located peripherally, and smaller and medium-sized ones in the central part (Figure 2A,C).

In some follicles, foamy colloids were observed, and occasionally the detachment of thyrocytes also occurred (Figure 2A,A′,C,C′).

In the thyroids of animals receiving feed supplemented with enriched lettuce, providing the recommended amount of iodine (groups 3–5), single large and medium-sized follicles were observed in the central part, whereas in other areas focal or multifocal detachment of thyrocytes into the follicular lumen was noted (Figure 2E,E′,G,G′,I,I′). In some follicles, foamy colloid was visible (Figure 2E,E′,G,G′).

The thyroids of rats from the groups receiving an increased iodine supply along with biofortified lettuce exhibited morphological changes depending on the form of iodine used. In the thyroid glands of rats fed lettuce enriched with potassium iodate (Group 6) as a dietary component, no clear division of follicles into peripheral and central zones was observed. In the central part of the glands, large and medium-sized follicles predominated, and multifocally, thyrocyte detachment into the follicular lumen was noted (Figure 2K,K′). The thyroid gland parenchyma of animals fed a diet supplemented with lettuce biofortified with iodoquinoline (Groups 7 and 8) showed large follicles in the peripheral zone, whereas in the central part small and medium-sized follicles were present, with occasional large ones (Figure 2M,O). In some follicles, thyrocyte detachment into the follicular lumen was observed, and the colloid exhibited a distinctly foamy structure (Figure 2M,M′,O,O′).

#### 3.4.2. Masson’s Trichrome Staining

In the thyroids of rats from all experimental groups, no changes were observed in the quantity or structure of collagen fibers in the interfollicular and interlobular tissue (Figure 2B,D,F,H,J,N,P). In contrast, mild fibrosis was observed in the thyroid gland of animals from group 6 (Figure 2L).

## 4. Discussion

In the present study, the bioavailability of iodine from lettuce biofortified with different forms of iodine was evaluated for the first time, and the effects of both adequate and excessive iodine intake (derived from this plant) on the organism of Wistar rats were analyzed. Iodine bioavailability is defined as the proportion of iodine that can be absorbed in the gastrointestinal tract, delivered to the bloodstream, and subsequently utilized for thyroid hormone synthesis or accumulated in body tissues [24,25].

### 4.1. Indicators of Iodine Status in Rats Fed Iodoquinoline-Biofortified Lettuce

#### 4.1.1. Iodine Excretion in Urine and Feces

One in vivo method for assessing the absorption of ingested iodine is the analysis of its excretion levels in urine and feces. Excess iodine (in inorganic form; I^−^) is primarily excreted via urine (approximately 90%) and, to a lesser extent, through feces and sweat [26]. Tonacchera et al. [8] demonstrated that biofortification of vegetables (lettuce, potatoes, carrots, and cherry tomatoes) with iodine through foliar feeding contributes to an increase (19.6%) in urinary iodine content in healthy volunteers. Kopeć et al. [27] and Rakoczy et al. [28] observed that rats fed a diet supplemented with iodine-biofortified lettuce (in the form of KI), through soil fertilization and foliar application, respectively, excreted less iodine in both urine and feces compared to rats fed the control diet. The iodine content in the feeds used in these experiments corresponded to its concentration in the basal diet.

In the experiment conducted by our team, it was found that the level of iodine excreted in urine and feces by rats was more dependent on the iodine content in the fortified lettuce than on the iodine form used for its biofortification. In animals from Groups 3–5, receiving lettuce supplying iodine in the same dose as the control diet, a decrease in iodine concentration in urine was observed, which aligns with findings by Kopeć et al. [27] and Rakoczy et al. [28]. This was presumably due to the biofortification process leading to an almost twofold increase (on average by 96.37%) in polyphenolic compounds content in the plant [17]. The most common polyphenolic compounds in lettuce (*Lactuca sativa* L.) are phenolic acids (mainly caffeic acid, chlorogenic acid, and their derivatives) and flavonoids (quercetin and kaempferol derivatives) [29,30]. These compounds may interact with iodine, forming complexes that can hinder its subsequent absorption in the intestines, thereby reducing its excretion via urine [31,32]. However, interactions between dietary polyphenols and iodine within plant-based matrices remain incompletely characterized. Nevertheless, in rats from Groups 4 and 5, which were fed diets supplemented with lettuce enriched with iodine in the form of iodoquinoline (8-hydroxy-7-iodo-5-quinolinesulfonic acid and 5,7-diiodo-8-quinolinol, respectively), an increase in iodine content was observed in the liver, heart, testes, and thyroid (in Group 4). This may indicate increased absorption and bioavailability of iodine from the gastrointestinal tract following consumption of lettuce biofortified with organic iodine forms. Additionally, it is likely that most of the available iodine was utilized for thyroid hormone synthesis, which might have contributed to reduced iodine excretion in urine as part of the organism’s effort to maintain iodine homeostasis in rats [33]. The thyroid hormone levels in the serum of rats from Groups 3–5 remained within the reference range established for this species. Conversely, the increased iodine content in the urine of rats from Groups 6–8 probably resulted from the iodine concentration in the feed being twice as high, exceeding the rats’ iodine requirements.

The increase in iodine excretion in feces observed in all groups fed diets with biofortified lettuce could result from changes in gut microbiota composition induced by iodine and the increased accumulation of bioactive compounds (mainly polyphenols, B vitamins, and vitamin C) in the plant [17]. In vivo studies have shown that supplementation with polyphenols [34], B vitamins [35], and vitamin C [36] can modulate gut microbiota in animal models, including increasing beneficial microorganisms and reducing harmful ones. Gut microbiota influences thyroid hormone levels by regulating iodine uptake, degradation, and the enterohepatic cycle. It should be noted that thyroid hormones themselves can modulate the composition and activity of gut microbiota, indicating mutual interactions within the gut–thyroid axis [37,38]. Moreover, studies suggest the presence of alternative mechanisms of thyroid hormone metabolism, where bacteria serve as their reservoir. Thyroxine (T4) conjugated with glucuronide can bind to bacteria, be stored by them, and released later for absorption. Unconjugated T4 may also bind to bacteria in the rat intestine. Meanwhile, *Escherichia coli* bacteria can accumulate triiodothyronine (T3) by strong binding with bacterial thyroid hormone-binding proteins [39]. Therefore, it is highly probable that in this case, the intestinal reservoir serves a protective function and allows the rat’s organism to maintain hormonal balance. However, this study did not assess the impact of consuming biofortified lettuce on the gut microbiota composition of rats. Furthermore, the relationship between gut microbiota and thyroid function remains insufficiently understood. Additionally, in animals from Groups 6–8, increased iodine levels in feces could also be due to the higher iodine intake in their diet.

#### 4.1.2. Iodine Accumulation in Selected Organs

The total iodine content in the human body is approximately 15–20 mg, of which 70–80% is located in the thyroid gland, while the remaining part is found in organs and tissues such as the liver, kidneys, heart, quadriceps femoris muscle, and adipose tissue [40]. Kirchgessner et al. [41] reported that the overall iodine concentration in the tissues of Sprague-Dawley rats fed a semi-synthetic diet with varying iodine content (in the form of KI) increased proportionally to the level of iodine intake. Piątkowska et al. [42] found that supplementation with raw carrots biofortified with iodine (as KI; via soil fertilization) did not cause significant changes in iodine content in the kidneys, liver, heart, and thigh muscle of Wistar rats. On the other hand, Rakoczy et al. [28] observed an increase in iodine levels in the tissues of rats fed a diet supplemented with iodine-enriched lettuce.

In our study, iodine content was analyzed in the thyroid gland, liver, kidneys, heart, and testes. The concentration of this element in these organs depended both on the form of iodine used to fortify the lettuce and on its amount in the feed. These organ-level differences indicate that the observed iodine enrichment results from a combination of enhanced bioavailability and modified post-absorptive handling, with increased thyroidal utilization on one hand, and altered short-term retention or slower clearance in metabolically active and excretory organs on the other.

In rats from Group 3, which received lettuce biofortified with inorganic iodine (KIO_3_) in their diet, a decrease in iodine concentration was observed in all examined organs. One potential reason for this outcome may be that the lettuce fortified with iodine in the form of KIO_3_ had the highest calcium and potassium contents, with increases of 31.12% and 11.99%, respectively, compared to the control lettuce [17]. A higher intake of calcium and potassium in the diet can adversely affect iodine bioavailability [25,32]. Furthermore, among Groups 3–5, the proportion of lettuce in the diet of rats from Group 3 was the highest (10.44 g kg^−1^) due to the lowest iodine content, as all diets were balanced for iodine concentration. Therefore, at an optimal iodine level in the diet, lettuce as a component contributed the highest levels of K and Ca, which could have further reduced iodine absorption. Moreover, iodine might have accumulated in larger amounts in other parts of the body, such as the skin or fur, which were not assessed in this study. In the experiment by Kirchgessner et al. [41], the highest iodine concentrations in rats, apart from the thyroid, were found in the skin, fur, and carcass.

In contrast, the increased iodine concentrations in the liver, heart, and testes of rats from Group 5 could have resulted from elevated levels of iron (16.00%), manganese (136.84%), and zinc (41.36%) in lettuce biofortified with 5,7-diiodo-8-quinolinol [17]. These trace elements may interact with iodine, influencing its absorption in the gastrointestinal tract [12,32]. Absorption can also be modified by competition between iodine and other elements or compounds present in the food matrix for the same transport mechanisms [31]. Conversely, a possible reason for the increased iodine content in the thyroid gland and other examined organs of rats from Group 4 may be the lower proportion of lettuce in the diet, which was associated with reduced polyphenol intake. These compounds, as mentioned earlier, can bind iodine and strongly inhibit its absorption [32]. Group 4 also showed a lower iodine content in the kidneys, presumably related to decreased urinary excretion of this element. In rats from Groups 7 and 8, increased iodine accumulation in all analyzed organs likely resulted from the higher iodine intake with the diet. Additionally, it may have been caused by the previously described factors, as in Groups 4 and 5.

### 4.2. Biochemical Parameters of Serum in Rats Fed Iodoquinoline-Biofortified Lettuce

#### 4.2.1. Thyroid-Related Hormones (T3, fT3, T4, fT4, TSH)

In the thyroid gland, tyrosyl residues of thyroglobulin are iodinated to form monoiodotyrosine (MIT) and subsequently diiodotyrosine (DIT). The coupling of two DIT molecules results in the formation of thyroxine (T4), while the coupling of DIT with MIT leads to the formation of triiodothyronine (T3) [43]. T3 is the biologically active hormone, whereas T4 is converted to T3 in peripheral tissues by deiodinases, which remove an iodine atom from the T4 molecule [12]. Thyroid hormones are transported in the bloodstream primarily bound to plasma proteins. However, only a small fraction of free (unbound) triiodothyronine (fT3) and thyroxine (fT4) is biologically active and capable of entering target cells to exert physiological effects [44].

Lee et al. [45] reported increased serum levels of T3 and T4 in Holstein dairy cows fed a diet supplemented with brown seaweed waste. In contrast, Franke et al. [46] did not observe statistically significant changes in serum T3 and T4 levels in dairy cows receiving either rapeseed meal or glucosinolate-free feed enriched with two different forms of iodine (potassium iodide or calcium iodate) at seven concentrations (0, 0.5, 1, 2, 3, 4, and 5 mg kg^−1^ dry matter). Only at the doses of 0.5 mg and 5 mg of iodine per kg of dry feed was the T4 level significantly lower than in the control group. Similar results were obtained by Aoe et al. [47], who investigated the effects of kelp (*Laminaria japonica*) consumption on thyroid hormone levels (T3, T4, and TSH) in overweight individuals. No increases in serum T3, T4, or TSH were observed after iodine supplementation at a dose of 1.03 mg/day derived from this seaweed.

In our study, thyroid hormone concentrations depended equally on the iodine dose in the diet and the form used to fortify the lettuce. The inclusion of lettuce enriched with iodoquinoline, providing iodine in the amount recommended for rats (Groups 4 and 5), did not result in significant changes in the levels of the assessed thyroid hormones. However, an increase in serum T4 levels was observed in animals from Groups 4 and 5, which may have resulted from improved iodine absorption from lettuce biofortified with organic forms of the element and, consequently, more efficient utilization of iodine in T4 synthesis. It can be speculated that the ratio of DIT to MIT was elevated, which favors T4 biosynthesis. In this way, euthyroid status is maintained despite increased iodine uptake by the gland [43,48], particularly given that T4 is the less biologically active form.

Different outcomes were noted in rats from Group 3, which were fed a diet containing lettuce fortified with potassium iodate. In this group, as with iodine concentrations in the analyzed organs, a reduction in thyroid hormone levels was recorded. The decrease in serum T3, T4, fT3, and fT4 levels in Group 3 rats may have resulted from the elevated content of antinutritional compounds in the iodate-enriched lettuce, which may inhibit the absorption and/or utilization of iodine after ingestion [49]. Furthermore, multiple interactions may occur between iodine and other elements present in the rats’ diet. As previously mentioned, the diet of this group had higher calcium and potassium levels, which could have affected iodine absorption in the gastrointestinal tract [17]. In general, food undergoes various chemical and physical processes after ingestion that may alter the amount of iodine ultimately reaching systemic circulation [50].

In rats from Groups 6–8, double the iodine intake led to increased thyroid gland stimulation and elevated serum concentrations of all analyzed thyroid hormones. Nevertheless, the values remained within the reference range for these animals, and TSH levels did not change. Although all thyroid hormone values remained within reference ranges, the significant increases in T4, fT4, T3 and fT3 at the supranutritional iodine intake, particularly in rats receiving iodoquinoline-biofortified lettuce, together with unchanged TSH, indicate a modest up-regulation of thyroid hormone production and/or peripheral T4-to-T3 conversion rather than overt hyperthyroidism. This pattern is consistent with an adaptive regulatory response to improved iodine supply, with increased functional activity of the thyroid but preserved central feedback control. Nevertheless, such shifts within the physiological range may still be of regulatory relevance under prolonged exposure or in vulnerable groups and therefore warrant further investigation. An exception was the serum fT3 and fT4 levels in Group 8 rats, which were at or slightly above the reference range. The diet of animals in this group contained the highest amount of polyphenolic compounds, and therefore likely also flavonoids. Therefore, one potential explanation for the elevated fT3 and fT4 levels could be the influence of flavonoids present in the lettuce biofortified with 5,7-diiodo-8-quinolinol on the binding of T4 to transthyretin. It has been demonstrated that synthetic flavonoids primarily disrupt this binding with transthyretin (TTR), rather than with thyroxine-binding globulin (TBG) or albumin [51]. In rodents, thyroid hormones are mainly bound to TTR, which limits the ability to compensate for impaired T4 binding to TTR, as is possible in humans. Given that flavonoids (synthetic) can act as strong competitors in the binding of thyroid hormones to TTR, a shift in thyroid hormone homeostasis seems likely if not inevitable in rats [52]. Moreover, rodents may exhibit physiological fluctuations in thyroid hormone levels in response to mild stress, dietary changes, or altered feeding conditions. When combined with the absence of thyroid fibrosis features, this suggests an adaptive rather than pathological nature of the observed changes. Turakulov et al. [53] demonstrated that short-term immobilization of rats causes a significant increase in the secretion of both T3 and T4, with peak serum concentrations occurring during double immobilization. Similarly, studies by Dauncey and Morovat [54] suggest a postprandial rise in the secretion of T3, T4, fT3, and fT4 from the thyroid gland in piglets, dependent on the energy value and nutritional composition of the ingested feed.

#### 4.2.2. Lipid Profile (HDL, LDL + VLDL, Total Cholesterol, Triglycerides)

Iodine, as the main component of thyroid hormones (T3, T4) and a key regulator of thyroid gland function, plays an essential role in its physiology. Consequently, dietary iodine intake affects the functional activity of the thyroid gland and, in turn, lipid metabolism in the serum. Thyroid hormones can influence all aspects of lipid metabolism, including synthesis, mobilization, and degradation, with degradation being more prominent than synthesis [55]. Some of the best-known effects of thyroid hormones on lipid metabolism include increased utilization of lipid substrates, enhanced synthesis and mobilization of triglycerides stored in adipose tissue, elevated levels of non-esterified fatty acids (NEFA), and increased lipoprotein lipase (LPL) activity [56].

In a study by Zhao et al. [57] evaluating the effects of both iodine deficiency and excess intake on lipid metabolism in Balb/c mice, a reduction in triglyceride (TG) levels in males and total cholesterol (TC) in females was observed in the iodine excess groups. On the other hand, Kopeć et al. [27] reported an increase in TC as well as low- and very-low-density lipoprotein (LDL + VLDL) levels in rats fed a diet containing lettuce biofortified with iodine in the form of KI, while high-density lipoprotein (HDL) and TG levels remained unchanged. However, in the study by Kopeć et al. [27], the iodine concentration in the feed was within the recommended range for rats. Waśniowska et al. [58] noted that administering red kale enriched with iodine in the form of 5,7-diiodo-8-quinolinol to rats contributed to a reduction in TC, HDL, and TG levels in their serum.

In our experiment, the serum levels of HDL cholesterol and TC in rats were more dependent on the amount of iodine in the diet than on the chemical form used to biofortify the lettuce. In contrast, the levels of LDL + VLDL cholesterol and TG were more influenced by the iodine form.

The observed decrease in HDL levels in the serum of animals from all groups receiving biofortified lettuce was likely the result of increased activity of cholesterol ester transfer protein (CETP) and hepatic lipase (HL), as these proteins are stimulated by thyroid hormones, which accelerate HDL metabolism. CETP transfers cholesterol esters from HDL2 to VLDL, IDL, and chylomicron remnants, while simultaneously transferring TG in the opposite direction—to HDL2. HDL2 is then hydrolyzed and converted into HDL3 by HL, which is the main lipolytic enzyme responsible for converting IDL to LDL and HDL2 to HDL3 [59,60]. Enhanced hydrolysis of triglyceride-rich HDL2 by HL and their conversion to HDL3 leads to the remodeling of HDL particles, which may result in decreased HDL levels [60]. In Groups 6–8, a twofold higher iodine dose provided with the diet resulted in a significant increase in thyroid hormone levels (with the exception of T4 in Group 6), which may have further enhanced the lipolysis process in HDL particles. This may explain the significantly lower HDL levels in the serum of rats from Groups 6–8 compared to those from Groups 3–5. It is well known that alterations in thyroid hormone levels, even fluctuations within the reference range, can have profound effects on lipid metabolism [61].

The decrease in LDL + VLDL concentrations in the serum of rats from Groups 3 and 5 was likely a consequence of the combined effects of polyphenols, other bioactive compounds (mainly B vitamins and vitamin C), and iodine (in an optimal amount) present in the lettuce. Both iodine, as a constituent of thyroid hormones, and the bioactive compounds in the biofortified lettuce can influence the activity of key metabolic enzymes and receptors involved in the regulation of LDL and VLDL synthesis. In rats, the primary mechanism by which thyroid hormones lower serum cholesterol is the induction of LDL receptor (LDL-R) expression in the liver, increasing cholesterol uptake and clearance. Thyroid hormones also stimulate the activity of 3-hydroxy-3-methylglutaryl-CoA reductase (HMGAR) and farnesyl diphosphate synthase (FDPS), thereby supporting cholesterol synthesis. Moreover, they reduce the hepatic level of apolipoprotein B100 (Apo B100), which leads to decreased production of LDL and VLDL [55]. Various types of natural food products, herbal preparations, and dietary supplements containing high concentrations of bioactive compounds (including polyphenols) have been shown to enhance LDL-R activity and inhibit HMGAR expression, thereby reducing LDL levels [62]. Caffeic acid and chlorogenic acid, administered to ICR mice, also demonstrated inhibitory effects on HMGAR [63]. In the liver, thyroid hormones increase the expression of the rat enzyme cholesterol 7α-hydroxylase (CYP7A1), which controls the first step in bile acid synthesis and indirectly regulates cholesterol biosynthesis and blood LDL levels [64,65]. In the study by Tan et al. [66], chlorogenic acid from green tea increased *Cyp7a1* mRNA expression in 129/Sv mice. The increase in LDL + VLDL levels in the serum of rats from Group 6 may represent a physiological response to the consumption of a diet containing lettuce biofortified with inorganic iodine (KIO_3_), in which the iodine content was twice as high as in the control diet.

The reduction in TC observed in all experimental groups (except for Group 2) was a result of decreased HDL levels in the serum and, in Groups 3 and 5, also a reduction in LDL + VLDL levels. Therefore, all the above-mentioned factors affecting lipoprotein levels may have contributed to the decrease in TC.

Furthermore, in the serum of rats from Group 5, which received lettuce biofortified with iodine in the form of 5,7-diiodo-8-quinolinol, a reduction in TG levels was noted, confirming the findings of Waśniowska et al. [58] for red kale enriched with the same form of iodine. However, in that study, rats received feed with a higher iodine content than the amount present in the diet of the animals from this group. A possible explanation for the TG reduction in Group 5 may be increased LPL activity resulting from the effects of polyphenols and iodine present in the lettuce biofortified with 5,7-diiodo-8-quinolinol. Thyroid hormones are known to reduce hepatic TG synthesis and secretion, as well as regulate their metabolism at the vascular endothelium level [56,67]. LPL plays a key role in TG metabolism by catalyzing their hydrolysis in plasma [68]. Notably, in vivo studies have shown that polyphenol-rich apple extract may reduce TG levels by increasing LPL activity in a model of Triton WR-1339-induced endogenous hyperlipidemia [69].

#### 4.2.3. Liver Enzymes (ALT, AST, ALP)

Thyroid hormones and thus iodine influence hepatocyte activity and liver metabolism. In turn, the liver plays a key role in the activation, deactivation, transport, and metabolism of thyroid hormones [70]. Alanine aminotransferase (ALT), aspartate aminotransferase (AST), and alkaline phosphatase (ALP) are enzymes present in the liver and serve as biomarkers of its damage [71].

Research by Guo et al. [7] showed that administering iodine (as KIO_3_) in drinking water at concentrations of 600, 1200, or 2400 µg L^−1^ to BALB/c mice caused a significant increase in serum ALT and AST levels. In our study, ALT, AST, and ALP activity depended both on the form of iodine used for lettuce biofortification and its amount (excluding Groups 5 and 8) in the feed. The reduction of ALT and ALP levels in the serum of rats from Groups 3–5 and 8, and Groups 3 and 4, respectively, may result from the hepatoprotective effects of bioactive compounds in lettuce. This effect can be attributed to their antioxidant properties and ability to stimulate the endogenous antioxidant defense system. Since oxidative stress is involved in almost all mechanisms of liver injury, it is assumed that the antioxidant properties of these compounds play a significant role in their liver-protective mechanism [72]. Studies indicate hepatoprotective properties of, among others, chlorogenic acid. This compound reduced the levels of ALT, AST, ALP, and bilirubin in the serum of rats with hepatotoxicity induced by methamphetamine and carbon tetrachloride [73,74]. Moreover, a probable cause of the decreased ALT concentration in the serum of rats from Group 8 may be the indirect effect of iodine excess on the reduction of glucose-alanine cycle activity. In this cycle, alanine is produced in muscles through pyruvate transamination and then transported to the liver, where it is converted back to pyruvate by an ALT-catalyzed reaction; pyruvate can then be used in gluconeogenesis [75,76]. Similarly, the increased level of this enzyme in the serum of animals from Group 6 could result from enhanced glucose-alanine cycle activity due to their consumption of twice the amount of iodine present in lettuce enriched with potassium iodate.

Meanwhile, the increased ALP activity in the serum of rats from Groups 6 and 7 may have been caused by altered absorption of other minerals, such as zinc and magnesium, due to the doubled iodine intake. The active site of ALP contains three metal ions: two Zn^2+^ and one Mg^2+^, which are essential for its enzymatic activity. Therefore, a proper Mg^2+^/Zn^2+^ ratio is necessary for the enzyme’s normal function [77].

In contrast, the increased serum AST levels observed in rats from Group 6 may be linked to enhanced activity of the malate-aspartate shuttle, triggered by the doubled iodine dose. This shuttle facilitates the transfer of reducing equivalents between the cytoplasm and mitochondria. AST catalyzes the conversion of L-aspartate and α-ketoglutarate into oxaloacetate and L-glutamate, a key step in regulating the NAD^+^/NADH ratio [75,78]. Excess iodine may influence cellular metabolism by intensifying this process, leading to increased AST release into the bloodstream.

#### 4.2.4. Digestive Enzymes (Amylase, Lipase, Elastase)

The addition of lettuce to the diet of rats in all experimental groups, including the group receiving non-biofortified lettuce, contributed to a decrease in serum amylase activity. This effect can be primarily attributed to the polyphenolic compounds present in lettuce. It has been demonstrated that the inhibition of α-amylase activity by polyphenols is strongly related to their molecular structure, as it results from binding interactions between these compounds and the enzyme [79]. The strength of interactions between polyphenolic compounds and α-amylase is probably dependent on the formation of hydrogen bonds between hydroxyl groups and the enzyme’s catalytic active site. Furthermore, hydrophobic forces between the aromatic rings of polyphenols and tryptophan residues of α-amylase may influence the strength of this interaction [80]. Nevertheless, in the context of polyphenol binding, a competitive mechanism between polysaccharides and α-amylase has also been suggested [81].

On the other hand, the increase in serum lipase concentration in rats consuming lettuce enriched with iodine at twice the recommended amount (Groups 6–8) could be an adaptive response to alterations in lipid metabolism caused by excessive iodine intake. This interpretation is supported by the absence of significant changes in fecal elastase levels across all experimental groups. Elastase is known for its high specificity to pancreatic function, and its concentration decreases only in cases of pancreatic damage or exocrine insufficiency. Furthermore, the lack of increased amylase activity supports the assumption that excess iodine did not induce a systemic inflammatory response of the pancreas, which typically manifests as a concurrent rise in multiple pancreatic enzyme activities [82].

Summarizing, the alterations observed in the lipid profile and serum enzyme activities appear to reflect the combined influence of iodine status and the bioactive compounds present in the fortified lettuce. Shifts in cholesterol fractions and triglycerides, particularly at the higher iodine intake, are consistent with findings that variations in iodine supply can modulate hepatic lipid metabolism through thyroid hormone–dependent pathways. At the same time, the improved lipid parameters and stable ALT, AST and ALP activities in rats receiving iodoquinoline-fortified lettuce likely result from higher levels of polyphenols and antioxidant micronutrients in these variants, which are known to support hepatic integrity and favorably influence lipid homeostasis. Overall, these biochemical responses seem to arise from the interaction between iodine availability and the phytochemical profile of the biofortified lettuce, rather than from iodine alone.

#### 4.2.5. Urea

The urea level, similar to amylase concentration, decreased in the serum of rats from all experimental groups (except Group 7). Since urea is the final product of nitrogen metabolism, its reduced concentration may indicate a decreased rate of amino acid catabolism and reduced nitrogen turnover in the organism [83]. However, the urea content in the serum of animals fed with non-biofortified lettuce was higher than in rodents receiving biofortified lettuce. Iodine present in the studied lettuce may have limited the activity of enzymes involved mainly in the rate-limiting steps of the urea cycle, such as carbamoyl phosphate synthetase I and argininosuccinate synthetase, as well as the enzyme catalyzing the final step of this cycle, without which urea cannot be produced—arginase I. It is known that the activity of various urea cycle enzymes can act as a regulatory factor for urea synthesis [84]. On the other hand, the activity of the key enzyme of this cycle—arginase I, can be modulated in vivo by changes in manganese concentration. Moreover, it is important to emphasize that metabolic regulation of the activity of enzymes in this cycle is species-specific and may depend on the type of diet to which a given organism is naturally adapted [85].

#### 4.2.6. Malondialdehyde

Importantly, a decrease in malondialdehyde (MDA) concentration was observed in the serum of animals from Groups 6 and 8. It cannot be excluded that the observed effect resulted from the antioxidant properties and synergistic action of both iodine and bioactive compounds present in the biofortified lettuce. As a result, this may have contributed to a reduction in oxidative stress in the rats’ bodies by neutralizing reactive oxygen species and limiting lipid peroxidation [1]. Consequently, the reduced intensity of lipid peroxidation may have led to a decrease in MDA levels, the end product of this process and an important biomarker, which in turn could contribute to minimizing oxidative damage to cellular membranes [86]. It is also worth noting that the lower iodine content in the biofortified lettuce required an increased proportion of this plant in the rats’ diet, which may have resulted in a higher intake of polyphenolic compounds.

### 4.3. Expression of Genes Involved in Iodine Metabolism and Thyroid Hormone Synthesis in Rats Fed Iodoquinoline-Biofortified Lettuce

In the present study, the mRNA expression levels of genes involved in iodine metabolism and thyroid hormone synthesis were also evaluated, including *Dio1*, *Dio2*, *Slc5a5*, and *Tpo* (encoding DIO1, DIO2, NIS, and TPO, respectively). DIO1 and DIO2 (type 1 and type 2 deiodinases) are responsible for the conversion of thyroxine (T4) to the active form triiodothyronine (T3), with DIO1 acting mainly in peripheral organs (e.g., liver and kidneys), thereby influencing circulating T3 levels, whereas DIO2 performs a local function within target tissues [87]. In contrast, TPO (thyroid peroxidase) and NIS (sodium-iodide symporter) are essential for thyroid hormone biosynthesis, participating in the iodination of tyrosyl residues in thyroglobulin and the transport of iodide into thyrocytes, respectively [88].

The observed increase in *Dio1* mRNA expression accompanied by a decrease in *Dio2* expression in the thyroid of rats receiving a diet supplemented with KIO_3_-biofortified lettuce providing iodine at the recommended level (Group 3) may indicate the activation of a compensatory transcriptional regulatory mechanism triggered by reduced availability of this element. Such adaptive regulation may constitute part of an intracellular homeostatic response aimed at maintaining physiological T3 concentrations within the thyroid, despite decreased DIO2 activity [89]. These observations are partially supported by previous findings. Gereben et al. [90] reported a decrease in *Dio2* expression in the thyroid of hypothyroid rats compared to euthyroid animals, while Lavado-Autric et al. [91] noted increased *Dio1* and *Dio2* mRNA levels in the thyroids of rats maintained on an iodine-deficient diet. The DIO1 enzyme is involved not only in the aforementioned conversion of T4 to T3 but also in the deiodination of rT3 and less active iodothyronines released from thyroglobulin, thereby supporting the recycling of iodine within follicular cells. This additional function may also account for the increased *Dio1* mRNA levels observed in Group 3 [92]. Simultaneously, the reduced iodine content in the thyroid may have limited the availability of T4 (the substrate) for DIO2, contributing to the down-regulation of its expression. It is worth noting that the physiological role of DIO2 in the thyroid gland is relatively limited compared to its activity in the central nervous system, brown adipose tissue, or skeletal muscles [93]. In both humans and rats, *Dio2* mRNA levels in the thyroid are relatively low, supporting the notion that, under conditions of slight thyroid hormone reduction (within the reference range), *Dio2* expression is down-regulated, and the predominant role in regulating intracellular T3 levels is assumed by DIO1 [90]. Such an expression pattern may suggest a shift in the primary site of T3 production control from local synthesis within the thyroid (*Dio2*) to peripheral tissues (*Dio1*), which are responsible for maintaining systemic hormone levels [94]. Furthermore, elevated *Slc5a5* mRNA expression in this group of animals may indicate an increased capacity of thyrocytes for iodide uptake, potentially supporting thyroid hormone biosynthesis and the up-regulation of *Dio1* expression. Despite the simultaneous up-regulation of *Slc5a5* and *Dio1*, the lack of changes in *Tpo* mRNA levels may result from insufficient activation of nuclear T3 receptors due to its limited availability in the cytoplasm [95]. Moreover, the transcriptional changes in these genes were not accompanied by significant differences in TSH levels, suggesting that the thyroid response was independent of the classical feedback regulation mechanism of the hypothalamic-pituitary-thyroid (HPT) axis and was limited to local adaptation related to iodine transport and metabolism [89].

By contrast, the increased mRNA levels of all analyzed genes in the thyroid of rats receiving lettuce biofortified with the same form of iodine but providing it at twice the recommended amount (Group 6) may have resulted from stimulation of glandular activity and enhanced transcriptional activity in response to mild iodine excess. The increased synthesis of thyroid hormones, observed alongside elevated expression of the *Slc5a5* and *Tpo* genes, leads to greater availability of the substrate (T4), which may induce higher expression of *Dio1* and *Dio2* within the thyroid gland. This effect may be a consequence of dietary modification and represent a potential cause of metabolic changes occurring in the thyroid. The increased mRNA levels of *Slc5a5* and *Tpo* may also be related to the higher presence of flavonoids in the tested plant. Lettuce predominantly contains various quercetin derivatives, including glycosidic forms structurally related to rutin (quercetin-3-O-rutinoside) [96]. The study conducted by Gonçalves et al. [97] demonstrated that this compound can significantly induce the expression of *Slc5a5* and *Tpo* genes in the thyroid of rats. Although rutin is not present in lettuce in substantial amounts, due to its shared aglycone and structural similarity, it may exhibit biological activity comparable to other quercetin derivatives. Therefore, flavonoids present in lettuce may be involved in the transcriptional regulation of these genes, potentially through analogous molecular pathways. Moreover, the observed up-regulation of *Tpo* and *Dio2* transcripts may, in part, result from improved systemic redox status, as evidenced by significantly reduced serum MDA levels in rats. A lower degree of lipid peroxidation may favor the activation of regulatory pathways dependent on the Nrf2 transcription factor, which indirectly supports *Tpo* expression by protecting key transcription factors such as TTF-1 and Pax-8 from oxidative damage and functional impairment [98,99]. In the case of *Dio2*, enhanced cellular reducing capacity may promote the activation of FoxO1—a redox-sensitive transcriptional regulator that directly enhances the expression of this gene [100].

Conversely, in the thyroids of rats fed with feed containing lettuce biofortified with 8-hydroxy-7-iodo-5-quinolinesulfonic acid (Group 7), the mildly increased iodine intake did not result in significant changes in *Tpo* and *Dio2* mRNA levels, suggesting no up-regulation of these genes. This was likely due to the fact that serum MDA concentrations remained comparable to those in the control group, which may have supported the maintenance of stable gene expression. On the other hand, in the group of animals fed with lettuce enriched with 5,7-diiodo-8-quinolinol (Group 8), the observed decrease in MDA levels might have potentially contributed to the up-regulation of *Tpo* and *Dio2*. However, the absence of this effect suggests that the regulation of these genes may also be influenced by factors other than the organism’s redox status, including the chemical structure of the iodine form used for biofortification. The higher content of 5,7-diiodo-8-quinolinol in the lettuce (195.7 µg kg^−1^) compared to 8-hydroxy-7-iodo-5-quinolinesulfonic acid (9.7 µg kg^−1^) may have influenced the composition and amount of secondary metabolites in the plant [17], which could, in turn, have altered the bioavailability of active compounds in the diet and indirectly modulated the transcriptional activity of *Tpo* and *Dio2*. The observed differences in *Dio1*, *Dio2*, *Slc5a5*, and *Tpo* gene expression among the groups depend not only on the amount of dietary iodine but also on changes in the plant’s chemical profile resulting from the biofortification process. It is also possible that these effects are mediated by epigenetic mechanisms, such as histone modifications or promoter region methylation, which influence the accessibility of DNA sequences to transcription factors [101].

### 4.4. Histomorphological Evaluation of the Thyroid Gland in Rats Fed Iodoquinoline-Biofortified Lettuce

Thyroid glands of rats from the control group (Group 1) and the group receiving a diet supplemented with non-biofortified lettuce (Group 2; iodine was supplied via a mineral mix) showed no pathological changes, confirming normal gland function under adequate iodine supply. Intact follicular structure and physiological distribution of collagen fibers indicated the absence of fibrosis and inflammatory processes.

Groups of animals fed lettuce biofortified with potassium iodate (Group 3), 8-hydroxy-7-iodo-5-quinolinesulfonic acid (Group 4), or 5,7-diiodo-8-quinolinol (Group 5), supplying iodine at the recommended dose, exhibited the presence of foamy colloid and focal detachment of thyrocytes, which may indicate activation of the thyroid’s secretory function. The lack of fibrosis signs and preserved follicular architecture suggest that the tested iodine forms were well tolerated by the gland and did not cause degenerative changes.

Conversely, histological changes observed in the thyroids of rats fed diets containing lettuce biofortified with potassium iodate (Group 6), 8-hydroxy-7-iodo-5-quinolinesulfonic acid (Group 7), or 5,7-diiodo-8-quinolinol (Group 8), providing twice the recommended iodine amount, may reflect differing biological activities of the individual compounds, influencing the gland’s structure and potentially its function. Group 6 showed mild perivascular and interlobular fibrosis, which may indicate early adaptive processes or thyroid responses to increased iodine levels. In Group 8, no significant fibrosis was observed despite thyrocyte detachment and presence of foamy colloid, suggesting a greater capacity to maintain homeostasis under higher iodine intake. Overall, these observations point to increased secretory activity and adaptive processes in the thyroid gland, without signs of permanent structural damage [102].

From a histopathological perspective, the follicular enlargement, foamy colloid and focal thyrocyte detachment observed in several groups represent changes typically associated with increased functional activity of the thyroid gland rather than structural impairment. Enlarged follicles with vacuolated (foamy) colloid are characteristic of accelerated iodination and colloid resorption during heightened hormone synthesis, while limited thyrocyte detachment is frequently noted under conditions of intensified colloid processing. Importantly, these alterations occurred without epithelial atypia, inflammatory infiltration or fibrotic remodeling, indicating preserved structural integrity and supporting the interpretation that the thyroid responded adaptively to differences in iodine availability rather than undergoing pathological overstimulation.

### 4.5. Strengths, Limitations, and Potential Applications of the Study

The present study has several notable strengths. It is the first in vivo assessment of iodine bioavailability from lettuce biofortified with iodoquinolines, evaluating its effects on thyroid hormone synthesis, biochemical parameters, gene expression, and thyroid histomorphology. Within the study, both recommended and double dietary iodine doses were compared, allowing evaluation of adequate as well as mildly excessive intake. Additionally, a multidimensional approach combining molecular, biochemical, and histological analyses enables a comprehensive assessment of iodine metabolism and thyroid function.

Nevertheless, certain limitations should be considered. The study was conducted on Wistar rats, which may restrict direct extrapolation of the results to humans due to species-specific differences in iodine metabolism. Moreover, the intervention with the tested lettuce was short-term, precluding assessment of potential effects of long-term consumption. It should also be noted that the lettuce matrix itself may contribute to some of the observed effects. Variations in fiber, polyphenols, vitamins and other nutrients across the fortified variants may modulate metabolic and endocrine outcomes beyond the direct effects of iodine and interact with thyroid hormone–dependent processes. The potential matrix-related influences have therefore been taken into account in the interpretation of the study findings.

Despite these limitations, the findings of this study have significant implications and potential practical applications. They indicate the safety and efficacy of plant-based biofortification to increase dietary iodine intake, including the use of organic iodine forms, which showed higher tissue bioavailability and efficiency of thyroid hormone synthesis. The results may inform the development of future nutritional strategies and functional foods, particularly for populations at risk of iodine deficiency. Furthermore, the generated data provide valuable experimental evidence supporting the design of human clinical trials and may contribute to expanding scientific knowledge on the interactions between iodine, dietary bioactive compounds, and thyroid function.

## 5. Conclusions

In summary, this study demonstrated that iodine delivered through lettuce biofortified with iodoquinolines was bioavailable and effectively utilized in vivo in Wistar rats. The organic iodine forms (8-hydroxy-7-iodo-5-quinolinesulfonic acid and 5,7-diiodo-8-quinolinol) showed superior bioavailability compared to the inorganic form (potassium iodate), as evidenced by greater tissue accumulation and enhanced thyroid hormone synthesis. At a nutritionally adequate intake, the biofortified lettuce did not significantly affect thyroid hormone concentrations, thyroid-related gene expression (*Dio1*, *Dio2*, *Slc5a5*, *Tpo*), or thyroid morphology. Supranutritional iodine intake increased T4, fT4, T3, and fT3 levels, but all hormone concentrations remained within the physiological range. Importantly, even at twice the recommended iodine dose, no elevation in liver enzyme activity (ALT, AST, ALP), amylase, or urea levels was observed. Moreover, the reduction in MDA concentrations indicates lower oxidative stress. Taken together, these findings support the absence of hepatotoxic or broader systemic adverse effects in vivo.

To the best of our knowledge, this is the first study to intentionally exceed the recommended dietary iodine intake using a plant-based matrix enriched via biofortification. These results confirm the nutritional safety and efficacy of iodine biofortification, justifying the need for further research, including human nutritional clinical trials to validate its safety and practical applicability. Future studies should also investigate the long-term metabolic consequences of sustained consumption of biofortified vegetables.

## 6. Patents

We applied the method of the biofortification of vegetables in iodine cultivated using traditional, soilless, and hydroponic methods and the use of 8-hydroxy-7-iodo-5-quinolinesulfonic acid and 5,7-diiodo-8-quinolinol for biofortification of vegetables with iodine. These are patent application number P.443218 and P.443221, respectively, for the compounds (Polish Patent Office; 21 December 2022).

## Figures and Tables

**Table 1:** Composition of experimental diets.

Ingredient [g kg^−1^]	Diet #1	Diet #2	Diet #3	Diet #4	Diet #5	Diet #6	Diet #7	Diet #8
Corn starch	532.486	526.586	524.866	531.196	526.656	517.246	529.906	520.826
Casein	200.00	200.00	200.00	200.00	200.00	200.00	200.00	200.00
Saccharose	100.00	100.00	100.00	100.00	100.00	100.00	100.00	100.00
Soybean oil	70.00	70.00	70.00	70.00	70.00	70.00	70.00	70.00
Fiber	50.00	48.10 ^1^	47.18 ^2^	49.52 ^3^	48.03 ^4^	44.36 ^5^	49.04 ^6^	46.06 ^7^
Mineral mix	35.00	35.00 ^8^	35.00 ^9^	35.00 ^9^	35.00 ^9^	35.00 ^9^	35.00 ^9^	35.00 ^9^
Vitamin mix ^10^	10.00	10.00	10.00	10.00	10.00	10.00	10.00	10.00
Choline chloride	2.50	2.50	2.50	2.50	2.50	2.50	2.50	2.50
*tert*-butylhydroquinone	0.014	0.014	0.014	0.014	0.014	0.014	0.014	0.014
Lettuce ^11^:								
Unbiofortified (control)	-	7.80	-	-	-	-	-	-
Biofortified with potassium iodate	-	-	10.44	-	-	20.88	-	-
Biofortified with 8-hydroxy-7-iodo-5-quinolinesulfonic acid	-	-	-	1.77	-	-	3.54	-
Biofortified with 5,7-diiodo-8-quinolinol	-	-	-	-	7.80	-	-	15.60

Description of experimental diets: Diet #1—the AIN-93G (control) diet; Diet #2—the diet containing non-biofortified lettuce, with KI from the mineral mix providing iodine in the amount recommended for the AIN-93G diet; Diets #3, #4, #5—the diets containing biofortified lettuce, providing iodine in the amount recommended for the AIN-93G diet; Diets #6, #7, #8—the diets containing biofortified lettuce, providing iodine in an amount twice that recommended for the AIN-93G diet. ^1234567^ Dietary fiber content reduced by the amount of fiber from biofortified or control lettuce, respectively, by: ^1^ 1.90 g; ^2^ 2.82 g, ^3^ 0.48 g, ^4^ 1.97 g, ^5^ 5.64 g, ^6^ 0.96 g, ^7^ 3.94 g; ^8^ The amount of iodine in the mineral mix was the same as in the AIN-93G diet; ^9^ The mineral mix did not contain iodine, in this diet, the source of iodine was biofortified lettuce; ^10^ Vitamin mix composition in accordance with the AIN-93G diet; ^11^ The diets included lyophilized lettuce.

**Table 2:** Iodine content in the urine and feces of rats in the second and seventh week of the experiment.

Group Number	Urine [µg dm^−3^]		Feces [µg kg^−1^ d.w.]	
	Second Week	Seventh Week	Second Week	Seventh Week
Group #1 (Control)	854.59 ± 24.81 ^d^	856.25 ± 19.92 ^d^	827.47 ± 31.01 ^a^	826.02 ± 71.22 ^a^
Group #2	864.49 ± 13.99 ^d^	873.41 ± 14.88 ^de^	855.64 ± 22.93 ^a^	851.25 ± 66.13 ^a^
Group #3	635.04 ± 9.86 ^b^	588.84 ± 7.74 ^b^*	965.91 ± 67.72 ^b^	965.75 ± 70.15 ^b^
Group #4	757.51 ± 12.41 ^c^	529.52 ± 10.19 ^a^*	1030.54 ± 16.22 ^b^	1061.51 ± 64.77 ^b^
Group #5	605.05 ± 6.14 ^a^	610.07 ± 5.87 ^c^	1029.06 ± 40.47 ^b^	1011.45 ± 58.29 ^b^
Group #6	940.40 ± 33.47 ^e^	888.92 ± 9.60 ^e^*	1240.73 ± 41.30 ^d^	1358.88 ± 13.11 ^c^*
Group #7	1087.66 ± 15.71 ^f^	1032.51 ± 10.04 ^g^*	1169.80 ± 9.21 ^c^	1364.99 ± 55.18 ^c^*
Group #8	945.39 ± 5.36 ^e^	912.06 ± 4.37 ^f^*	1215.74 ± 46.68 ^cd^	1338.09 ± 21.60 ^c^*

Results are presented as mean ± standard deviation (n = 7). Different letters next to the values in the columns indicate significant differences between groups (*p* ≤ 0.05), while asterisks denote differences compared to the second week within the same groups (*p* ≤ 0.05); d.w.—dry weight. Description of experimental groups: **Group #1**—rats fed the AIN-93G (control) diet; **Group #2**—rats fed the AIN-93G diet with the addition of lyophilized non-biofortified lettuce, with KI from the mineral mixture providing iodine in the amount recommended for this diet; **Group #3**, **#4**, **#5**—rats fed the AIN-93G diet with the addition of lyophilized iodine-biofortified lettuce in the form of potassium iodate (Group #3), 8-hydroxy-7-iodo-5-quinolinesulfonic acid (Group #4), and 5,7-diiodo-8-quinolinol (Group #5), providing iodine in the amount recommended for this diet; **Group #6**, **#7**, **#8**—rats fed the AIN-93G diet with the addition of lyophilized iodine-biofortified lettuce in the form of potassium iodate (Group #6), 8-hydroxy-7-iodo-5-quinolinesulfonic acid (Group #7), and 5,7-diiodo-8-quinolinol (Group #8), providing iodine in an amount twice that recommended for this diet.

**Table 3:** Iodine content in selected organs of rats.

Group Number	Thyroid [mg kg^−1^ d.w.]	Liver [µg kg^−1^ d.w.]	Kidneys [µg kg^−1^ d.w.]	Heart [µg kg^−1^ d.w.]	Testes [µg kg^−1^ d.w.]
Group #1 (Control)	1716.20 ± 82.54 ^b^	115.20 ± 11.20 ^b^	190.80 ± 4.84 ^c^	54.98 ± 2.98 ^a^	155.75 ± 11.31 ^b^
Group #2	1797.98 ± 110.29 ^bc^	118.94 ± 7.00 ^b^	187.14 ± 2.77 ^c^	45.17 ± 6.34 ^a^	155.83 ± 9.20 ^b^
Group #3	1532.36 ± 34.02 ^a^	85.87 ± 9.69 ^a^	129.91 ± 3.95 ^a^	51.06 ± 4.22 ^a^	97.91 ± 3.75 ^a^
Group #4	1840.78 ± 57.63 ^c^	156.02 ± 6.14 ^c^	152.39 ± 9.73 ^b^	74.23 ± 6.83 ^b^	171.07 ± 5.64 ^c^
Group #5	1825.34 ± 68.90 ^bc^	153.24 ± 3.53 ^c^	189.79 ± 8.20 ^c^	86.19 ± 5.39 ^c^	199.27 ± 8.88 ^d^
Group #6	1778.71 ± 15.38 ^bc^	110.35 ± 5.91 ^b^	183.19 ± 5.34 ^c^	68.18 ± 8.61 ^b^	106.18 ± 5.19 ^a^
Group #7	1975.56 ± 10.25 ^d^	175.85 ± 2.42 ^d^	208.82 ± 8.37 ^d^	96.66 ± 2.02 ^d^	214.87 ± 1.74 ^e^
Group #8	1961.46 ± 71.66 ^d^	192.58 ± 4.77 ^e^	219.01 ± 3.15 ^d^	94.15 ± 7.10 ^cd^	224.99 ± 12.05 ^e^

Results are presented as mean ± standard deviation (n = 7). Values in columns marked with a different letter are significantly different at *p* ≤ 0.05; d.w.—dry weight. Description of experimental groups: **Group #1**—rats fed the AIN-93G (control) diet; **Group #2**—rats fed the AIN-93G diet with the addition of lyophilized non-biofortified lettuce, with KI from the mineral mixture providing iodine in the amount recommended for this diet; **Group #3**, **#4**, **#5**—rats fed the AIN-93G diet with the addition of lyophilized iodine-biofortified lettuce in the form of potassium iodate (Group #3), 8-hydroxy-7-iodo-5-quinolinesulfonic acid (Group #4), and 5,7-diiodo-8-quinolinol (Group #5), providing iodine in the amount recommended for this diet; **Group #6**, **#7**, **#8**—rats fed the AIN-93G diet with the addition of lyophilized iodine-biofortified lettuce in the form of potassium iodate (Group #6), 8-hydroxy-7-iodo-5-quinolinesulfonic acid (Group #7), and 5,7-diiodo-8-quinolinol (Group #8), providing iodine in an amount twice that recommended for this diet.

**Table 4:** Hormone levels associated with thyroid function in rats.

Group Number	Thyroxine (T4) [nmol L^−1^]	Free T4 (fT4) [pmol L^−1^]	Triiodothyronine (T3) [nmol L^−1^]	Free T3 (fT3) [pmol L^−1^]	Thyroid-Stimulating Hormone (TSH) [mIU L^−1^]
Group #1 (Control)	101.00 ± 5.46 ^b^	21.62 ± 2.81 ^b^	1.30 ± 0.03 ^b^	7.17 ± 0.18 ^b^	4.12 ± 0.15 ^ab^
Group #2	101.50 ± 6.17 ^b^	20.08 ± 1.89 ^b^	1.31 ± 0.06 ^b^	7.55 ± 0.53 ^b^	4.18 ± 0.19 ^ab^
Group #3	84.70 ± 2.88 ^a^	14.59 ± 1.01 ^a^	1.16 ± 0.04 ^a^	6.38 ± 0.16 ^a^	4.05 ± 0.11 ^a^
Group #4	111.35 ± 3.83 ^cd^	18.40 ± 2.45 ^b^	1.36 ± 0.11 ^b^	7.18 ± 0.29 ^b^	4.23 ± 0.26 ^ab^
Group #5	111.60 ± 7.02 ^cd^	19.55 ± 1.38 ^b^	1.23 ± 0.07 ^ab^	7.42 ± 0.45 ^b^	4.26 ± 0.17 ^ab^
Group #6	105.17 ± 1.51 ^bc^	25.66 ± 1.15 ^c^	1.97 ± 0.14 ^c^	8.53 ± 0.72 ^c^	4.02 ± 0.08 ^a^
Group #7	114.98 ± 4.92 ^d^	27.73 ± 1.93 ^c^	2.13 ± 0.05 ^d^	9.09 ± 0.63 ^c^	4.19 ± 0.14 ^ab^
Group #8	116.90 ± 7.40 ^d^	34.72 ± 1.90 ^d^	2.84 ± 0.09 ^e^	10.92 ± 0.44 ^d^	4.38 ± 0.09 ^b^

Results are presented as mean ± standard deviation (n = 7). Values in columns marked with a different letter are significantly different at *p* ≤ 0.05. Description of experimental groups: **Group #1**—rats fed the AIN-93G (control) diet; **Group #2**—rats fed the AIN-93G diet with the addition of lyophilized non-biofortified lettuce, with KI from the mineral mixture providing iodine in the amount recommended for this diet; **Group #3**, **#4**, **#5**—rats fed the AIN-93G diet with the addition of lyophilized iodine-biofortified lettuce in the form of potassium iodate (Group #3), 8-hydroxy-7-iodo-5-quinolinesulfonic acid (Group #4), and 5,7-diiodo-8-quinolinol (Group #5), providing iodine in the amount recommended for this diet; **Group #6**, **#7**, **#8**—rats fed the AIN-93G diet with the addition of lyophilized iodine-biofortified lettuce in the form of potassium iodate (Group #6), 8-hydroxy-7-iodo-5-quinolinesulfonic acid (Group #7), and 5,7-diiodo-8-quinolinol (Group #8), providing iodine in an amount twice that recommended for this diet.

**Table 5:** Lipid profile of rat serum.

Group Number	HDL Cholesterol [mg dL^−1^]	LDL + VLDL Cholesterol [mg dL^−1^]	Total Cholesterol [mg dL^−1^]	Triglycerides [mg dL^−1^]
Group #1 (Control)	59.98 ± 1.03 ^c^	17.08 ± 1.23 ^c^	77.06 ± 4.40 ^c^	230.48 ± 11.69 ^bc^
Group #2	57.99 ± 0.84 ^c^	16.26 ± 0.47 ^c^	74.25 ± 3.15 ^c^	230.70 ± 9.15 ^bc^
Group #3	45.16 ± 0.76 ^b^	14.59 ± 0.68 ^b^	59.75 ± 1.62 ^ab^	219.44 ± 3.08 ^b^
Group #4	47.35 ± 1.29 ^b^	16.78 ± 0.56 ^c^	64.13 ± 1.98 ^b^	237.15 ± 12.49 ^c^
Group #5	46.78 ± 1.77 ^b^	12.30 ± 0.81 ^a^	59.08 ± 2.37 ^ab^	167.98 ± 8.61 ^a^
Group #6	40.20 ± 2.18 ^a^	20.50 ± 1.14 ^d^	60.70 ± 4.01 ^ab^	216.20 ± 8.88 ^b^
Group #7	42.00 ± 0.62 ^a^	17.06 ± 0.97 ^c^	59.06 ± 3.35 ^a^	225.70 ± 6.14 ^bc^
Group #8	40.80 ± 1.57 ^a^	15.87 ± 1.11 ^bc^	56.67 ± 1.70 ^a^	238.27 ± 4.47 ^c^

Results are presented as mean ± standard deviation (n = 7). Values in columns marked with a different letter are significantly different at *p* ≤ 0.05. Description of experimental groups: **Group #1**—rats fed the AIN-93G (control) diet; **Group #2**—rats fed the AIN-93G diet with the addition of lyophilized non-biofortified lettuce, with KI from the mineral mixture providing iodine in the amount recommended for this diet; **Group #3**, **#4**, **#5**—rats fed the AIN-93G diet with the addition of lyophilized iodine-biofortified lettuce in the form of potassium iodate (Group #3), 8-hydroxy-7-iodo-5-quinolinesulfonic acid (Group #4), and 5,7-diiodo-8-quinolinol (Group #5), providing iodine in the amount recommended for this diet; **Group #6**, **#7**, **#8**—rats fed the AIN-93G diet with the addition of lyophilized iodine-biofortified lettuce in the form of potassium iodate (Group #6), 8-hydroxy-7-iodo-5-quinolinesulfonic acid (Group #7), and 5,7-diiodo-8-quinolinol (Group #8), providing iodine in an amount twice that recommended for this diet.

**Table 6:** Other biochemical parameters related to proper functioning of the rat organism.

**Group Number**	**Alanine** **Aminotransferase** **[U L^−1^]**	**Aspartate** **Aminotransferase** **[U L^−1^]**	**Alkaline** **Phosphatase** **[U L^−1^]**	**Urea** **[mg dL^−1^]**
Group #1 (Control)	40.70 ± 2.25 ^c^	95.53 ± 1.71 ^ab^	16.20 ± 0.97 ^c^	36.90 ± 1.65 ^e^
Group #2	40.90 ± 1.4 ^c^	97.75 ± 4.54 ^ab^	14.57 ± 1.58 ^bc^	34.90 ± 0.61 ^d^
Group #3	33.99 ± 1.08 ^ab^	100.40 ± 8.66 ^bc^	12.29 ± 0.40 ^a^	32.70 ± 1.23 ^c^
Group #4	36.19 ± 3.63 ^b^	95.80 ± 6.75 ^ab^	14.20 ± 0.49 ^b^	26.27 ± 0.58 ^a^
Group #5	32.36 ± 0.66 ^a^	90.44 ± 2.53 ^a^	16.00 ± 0.62 ^c^	27.36 ± 0.80 ^a^
Group #6	50.37 ± 2.82 ^d^	106.60 ± 3.95 ^c^	23.60 ± 1.33 ^e^	34.13 ± 1.47 ^cd^
Group #7	41.06 ± 0.71 ^c^	103.72 ± 1.62 ^bc^	18.14 ± 0.51 ^d^	35.78 ± 1.06 ^de^
Group #8	35.23 ± 1.19 ^ab^	97.13 ± 5.30 ^ab^	16.00 ± 1.08 ^c^	29.23 ± 0.94 ^b^
**Group Number**	**Amylase** **[U L^−1^]**	**Lipase** **[U L^−1^]**	**Elastase** **[ng g^−1^d.w.]**	**Malondialdehyde [nmol mL^−1^]**
Group #1 (Control)	681.57 ± 17.15 ^d^	187.95 ± 9.43 ^ab^	1610.35 ± 37.65 ^a^	3.71 ± 0.17 ^bc^
Group #2	554.09 ± 19.01 ^b^	183.17 ± 6.97 ^a^	1627.77 ± 18.78 ^a^	3.52 ± 0.23 ^abc^
Group #3	581.72 ± 8.71 ^bc^	199.14 ± 6.52 ^b^	1615.24 ± 77.14 ^a^	3.65 ± 0.21 ^bc^
Group #4	384.03 ± 13.46 ^a^	178.29 ± 4.67 ^a^	1565.59 ± 35.74 ^a^	3.84 ± 0.14 ^c^
Group #5	588.87 ± 9.52 ^c^	189.56 ± 5.11 ^ab^	1613.19 ± 102.99 ^a^	3.54 ± 0.26 ^abc^
Group #6	601.00 ± 18.78 ^c^	259.32 ± 10.30 ^d^	1628.57 ± 48.69 ^a^	3.20 ± 0.22 ^a^
Group #7	584.25 ± 20.00 ^c^	226.29 ± 12.41 ^c^	1625.43 ± 63.76 ^a^	3.46 ± 0.29 ^ab^
Group #8	607.20 ± 27.95 ^c^	221.79 ± 8.79 ^c^	1587.04 ± 52.26 ^a^	3.21 ± 0.23 ^a^

Results are presented as mean ± standard deviation (n = 7). Values in columns marked with a different letter are significantly different at *p* ≤ 0.05; d.w.—dry weight. Description of experimental groups: **Group #1**—rats fed the AIN-93G (control) diet; **Group #2**—rats fed the AIN-93G diet with the addition of lyophilized non-biofortified lettuce, with KI from the mineral mixture providing iodine in the amount recommended for this diet; **Group #3**, **#4**, **#5**—rats fed the AIN-93G diet with the addition of lyophilized iodine-biofortified lettuce in the form of potassium iodate (Group #3), 8-hydroxy-7-iodo-5-quinolinesulfonic acid (Group #4), and 5,7-diiodo-8-quinolinol (Group #5), providing iodine in the amount recommended for this diet; **Group #6**, **#7**, **#8**—rats fed the AIN-93G diet with the addition of lyophilized iodine-biofortified lettuce in the form of potassium iodate (Group #6), 8-hydroxy-7-iodo-5-quinolinesulfonic acid (Group #7), and 5,7-diiodo-8-quinolinol (Group #8), providing iodine in an amount twice that recommended for this diet.

## Data Availability

The datasets generated and/or analyzed during the current study are available from the corresponding authors on reasonable request.

## Supplementary Materials

The following supporting information can be downloaded at: https://www.mdpi.com/article/10.3390/nu18010036/s1, Table S1: The iodine content in experimental rat feed.
