# Supplementary material for: Iodoquinoline-Biofortified Lettuce as a Safe and Bioavailable Dietary Iodine Source: In Vivo Study in Rats
Source: Nutrients. 2025 Dec 21;18(1):36. doi: 10.3390/nu18010036 (PMC12787694; doi:10.3390/nu18010036)
Supplement: Supplementary file 1 [file nutrients-18-00036-s001.zip › nutrients-4026457-supplementary.pdf]

Supplementary Materials

Iodoquinoline-Biofortified Lettuce as a Safe and Bioavailable Dietary Iodine Source: *In Vivo* Study in Rats

Agnieszka Dyląg [1,*], Piotr Pawlicki [2], Anna Gałuszka [2], Sylwester Smoleń [3] and Aneta Koronowicz [1,*]

[1] Department of Human Nutrition and Dietetics, Faculty of Food Technology, University of Agriculture in Krakow, ul. Balicka 122, 31-149 Krakow, Poland; agnieszka.dylag@poczta.fm (A.D.); aneta.koronowicz@urk.edu.pl (A.K.)

[2] Department of Basic Sciences, Faculty of Veterinary Medicine, University of Agriculture in Krakow, Redzina 1c, 30–248 Krakow, Poland; piotr.pawlicki@urk.edu.pl (P.P.); anna.galuszka@urk.edu.pl (A.G.)

[3] Department of Plant Biology and Biotechnology, Faculty of Biotechnology and Horticulture, University of Agriculture in Krakow, al. Mickiewicza 21, 31-120 Krakow, Poland; sylwester.smolen@urk.edu.pl (S.S.)

* Correspondence: agnieszka.dylag@poczta.fm (A.D.); aneta.koronowicz@urk.edu.pl (A.K.)

Table S1. The iodine content in experimental rat feed.

Diet number	Iodine content [mg kg^{-1} d.w.]
Diet #1	0.23 ± 0.02[a]
Diet #2	0.23 ± 0.01[a]
Diet #3	0.23 ± 0.01[a]
Diet #4	0.26 ± 0.01[a]
Diet #5	0.25 ± 0.02[a]
Diet #6	0.44 ± 0.03[b]
Diet #7	0.49 ± 0.01[b]
Diet #8	0.48 ± 0.02[b]

Results are presented as mean ± standard deviation (n = 4). Values in columns marked with a different letter are significantly different at $p \leq 0.05$; d.w. – dry weight.

Description of experimental diets: **Diet #1** – the AIN-93G diet (control); **Diet #2** – the diet containing lyophilized non-biofortified lettuce, with KI from the mineral mixture providing iodine in the amount recommended for the AIN-93G diet; **Diet #3, #4, #5** – the diet containing lyophilized lettuce biofortified with iodine in the form of potassium iodate (Diet #3), 8-hydroxy-7-iodo-5-quinolinesulfonic acid (Diet #4), and 5,7-diiodo-8-quinolinol (Diet #5), providing iodine in the amount recommended for the AIN-93G diet; **Diet #6, #7, #8** – the diet containing lyophilized lettuce biofortified with iodine in the form of potassium iodate (Diet #6), 8-hydroxy-7-iodo-5-quinolinesulfonic acid (Diet #7), and 5,7-diiodo-8-quinolinol (Diet #8), providing iodine in an amount twice that recommended for the AIN-93G diet.
